# Neural Correlates of Inhibitory Control in Impulsivity Traits in Non-Ecological Human–Computer Tasks: An ALE Meta-Analysis

**DOI:** 10.3390/brainsci16060609

**Published:** 2026-06-03

**Authors:** Chiara Noferini, Gioele Gavazzi, Fabio Giovannelli, Chiara Puddu, Mario Mascalchi, Massimo Cincotta, Liberatore Tramontano, Carlo Cavaliere, Maria Pia Viggiano

**Affiliations:** 1 Department of Physics and Astronomy, University of Florence, Via Giovanni Sansone 1, Sesto Fiorentino, 50019 Florence, Italy; chiara.noferini@unifi.it; 2 Department of Neuroscience, Psychology, Drug Research, Child Health (NEUROFARBA), University of Florence, 50139 Florence, Italy; gioele.gavazzi@unifi.it (G.G.); fabio.giovannelli@unifi.it (F.G.); 3 Studio di Psicologia Frammenti, Via Porta Al Borgo, 8, 51100 Pistoia, Italy; chiarapuddupsico@gmail.com; 4 “Mario Serio” Department of Experimental and Clinical Biomedical Sciences, University of Florence, 50134 Florence, Italy; mario.mascalchi@unifi.it; 5 Unit of Neurology of Florence, Central Tuscany Local Health Authority, 50143 Firenze, Italy; massimo.cincotta@uslcentro.toscana.it; 6 IRCCS SYNLAB SDN, 80143 Naples, Italy; liberatore.tramontano@synlab.it (L.T.); carlo.cavaliere@synlab.it (C.C.)

**Keywords:** SST, Go-Nogo, cognitive control, meta-analysis, impulsivity, prefrontal cortex

## Abstract

**Highlights:**

**What are the main findings?**

- Both impulsive and non-impulsive individuals recruit a common subcortical–prefrontal network during response inhibition in computerized paradigms.
- Impulsive individuals show different recruitment of the r-MFG and r-SFG compared to non-impulsive individuals.

**What are the implications of the main findings?**

- Refine neural models of impulsivity by dissociating shared versus trait-sensitive inhibition mechanisms.
- Suggest potential relevance for environments involving frequent interaction with artificial systems, with possible implications for adaptive technologies that support decision-making.

**Abstract:**

**Background/Objectives**: Response inhibition is the capacity to restrain impulsive actions, representing a pivotal facet of cognitive control. Although several studies report a dynamic relationship between impulsivity and inhibitory control, the neural mechanisms remain unclear. The aim of the present study is to explore neural correlates of response inhibition as a function of impulsive personality traits. **Methods**: For this purpose, two groups of fMRI studies conducted on subjects with and without impulsive traits were compared. The selected studies were included based on both the impulsivity levels and the performance of the subjects in inhibitory human–computer tasks (e.g., Go/No-go, Stop-signal). This was done to minimize potential differences due to samples’ performances. Neuroimaging data were analyzed with an Activation Likelihood Estimation (ALE) meta-analysis approach. **Results**: Results reveal highly congruent clusters encompassing subcortical and prefrontal brain regions in both impulsive and non-impulsive subjects, albeit with subtle distinctions. Specifically, a direct contrast highlighted different activity in the right Middle and Superior Frontal Gyrus during inhibition tasks in the impulsive group. **Conclusions**: These findings deepen our comprehension of the neural mechanisms governing inhibitory control in human impulsivity. Understanding such mechanisms is increasingly relevant in today’s world, where frequent interactions with artificial systems may challenge or modulate inhibitory control, with potential implications for everyday behavior.

## 1. Introduction

In an ever-changing world, the ability to modulate our behavior is crucial. Its regulation depends on higher-order mechanisms collectively referred to as cognitive control, which enables the flexible coordination of thoughts and actions according to internal goals and external environmental demands [1]. Throughout the process of behavioral regulation, an efficient inhibitory mechanism is crucial to ensure the suppression of automatic but intrusive and/or non-adaptive behavioral, emotional and cognitive responses within a specific context [2].

Cognitive control operates by means of two modes of response inhibition: Proactive and Reactive Inhibitory Control. Proactive Inhibitory Control is a top-down process driven by higher cognitive systems based on anticipatory preparation for the suppression of a specific response, whereas Reactive Inhibitory Control refers to the suppression of a motor response that is already ongoing and is a bottom-up process driven by intervening stimuli that enable the avoidance of unpredictable events [3,4,5,6].

The neural correlates of inhibitory control have been widely investigated (see, for example, [7,8,9,10,11,12,13,14]).

At rest, the system is maintained in equilibrium by two opposing forces: (i) the excitation of the Thalamus and the *salience network* modulated by the internal level of vigilance, and (ii) the inhibition exerted by the Globus pallidus on the motor system [10,15,16,17,18,19].

In particular, during the inhibitory proactive phase, the insular region and the red nucleus sustain the excitatory part, while the inhibitory component is driven by the Inferior Frontal Gyrus, which, probably through the caudate and subthalamic nuclei, acts on the Thalamus and the Globus pallidus. During reactive inhibition, the inhibitory force that is already exerted at the baseline is not sufficient, but further involvement of the Superior Frontal Gyrus and the Middle Frontal Gyrus is needed [5,19,20,21]. The interaction of these two inhibitory phases is monitored by the Anterior Cingulate Cortex which, communicating with parietal and prefrontal regions, updates and guides the system based on external sensory input. This coordination allows the system to switch from the proactive to the reactive phase as needed [4,5,20,22,23,24,25,26,27,28].

Furthermore, it may occur that the inhibitory regulation is overloaded by excessive salience attributed to external stimuli or internal factors. For instance, this is typically seen in psychopathological conditions implying a deficit in inhibitory processes, such as attention deficit hyperactivity disorder [29,30], drug addiction [31,32,33], pathological gambling [34,35], personality disorders [36,37], impulse control disorders and Parkinson’s disease [38,39]. In these clinical conditions, markedly impulsive behaviors are often observed [40,41].

Notwithstanding the multidimensional nature of impulsivity, the most commonly observed characteristics include a lack of action-planning, poor assessment of possible consequences and uncontrolled behaviors [40,42]. In healthy individuals it can identify temporary internal states (state impulsivity) as well as define an individual aspect of personality (trait impulsivity).

In general, trait impulsivity pervades the individual’s entire behavioral repertoire, leading to unproductivity that can interfere with career goals and social relationships [4,43], and it is often associated with aggression, propensity towards risky behavior, and a greater incidence of suicide [40,44,45]. Typically, trait impulsivity is measured through self-report questionnaires and the Barratt Impulsiveness Scale (BIS-11—[46]) is a 30-item questionnaire widely used for this purpose.

**Figure 1:**
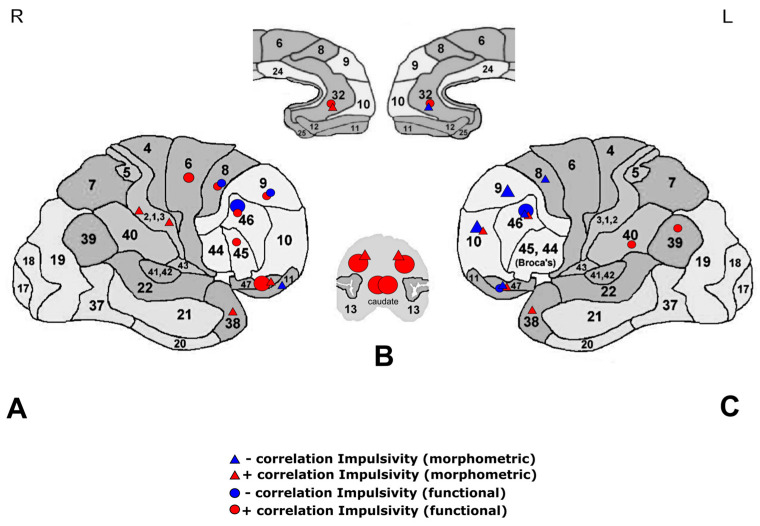
Representation of impulsivity-related brain regions. This figure depicts a schematic summary of the Brodmann areas (BA) positively or negatively correlated with impulsivity (red for positive correlations, blue for negative correlations) as found in the literature [47,50,54,56,57,58,59,60,61,63,64,65,66,67,68]. Panel (**A**) Side view and sagittal section of the BA brain chart. Panel (**B**) Coronal section of the BA brain chart. Panel (**C**) Side view and sagittal section of the BA brain chart. In each panel, morphometry studies are indicated by triangles and fMRI studies are indicated by circles. The size of the triangles and circles is proportional to the amount of studies that detect a correlation between that region and impulsivity.

Several studies have highlighted morphological and functional brain alterations in inhibitory control areas in people with the impulsivity trait, including frontal and prefrontal cortices, sensorimotor areas, the Anterior Cingulate Cortex and the caudate nucleus [4,47,48,49,50,51,52,53,54,55,56,57,58,59,60,61,62] (for a representation see Figure 1).

The inconsistency of previous findings reflects the use of different paradigms and heterogeneous theoretical frameworks. Given the significant role of impulsivity in several psychopathological conditions (e.g., substance addiction, gambling, aggressiveness, etc.), understanding the mechanisms underlying inhibitory control may contribute to the development of more effective approaches targeting this process, especially in increasingly complex and AI-driven environments that place substantial demands on cognitive control and decision-making.

The progressive transition toward AI-shaped technological devices has significantly accelerated the pace of events, exposing the brain to constant temporal pressure. In this context, inhibitory control may be particularly vulnerable, as its impairment has been associated with maladaptive impulsivity. Therefore, given the rising reliance on AI-driven machines, exploring impulsivity in non-ecological human–computer tasks is increasingly relevant for isolating pure computer interaction without contextual confounds.

In this context, the lack of unanimous agreement on the specific brain regions involved is a significant shortfall. To the best of our knowledge, there is currently no meta-analysis of the neural correlates associated with the impulsivity trait. Therefore, to fill this gap, we performed a meta-analysis of fMRI activation data from studies investigating inhibitory control in impulsive and non-impulsive subjects performing motor response inhibition tasks.

Specifically, in the analysis design, studies examining the two conditions, impulsive and non-impulsive subjects, were matched according to task reaction times. The *rationale* behind this choice is to control for differences in the task’s inhibitory demand, measurable through the reaction times. This reasoning is supported by the observation that successful inhibition, which is associated with faster reaction times, requires over-recruitment of higher regions of the prefrontal cortex, as demonstrated by Gavazzi et al. [19]. By matching subjects on reaction time, we aim to isolate the role of impulsivity in inhibitory control regardless of the variability in task performance. Consistent with our previous work [19], the same reaction-time-matching procedure was employed; yet, the present study builds upon the former by explicitly incorporating impulsivity as a variable of interest and directly assessing its contribution to inhibitory control in non-ecological settings.

To this purpose, we selected fMRI studies using exclusively non-ecological human–computer tasks—Go/NoGo and/or Stop Signal Task (SST)—assessing trait impulsivity through self-report tests. These data were meta-analyzed with the Activation Likelihood Estimation (ALE) to disentangle critical brain regions activated during an inhibitory process in impulsive subjects.

This exploration may have increasing relevance in contemporary environments where frequent interactions with artificial systems place continuous demands on inhibitory control and decision-making. Understanding how impulsivity modulates these processes may inform the design of adaptive systems aimed at reducing maladaptive impulsive behaviors.

## 2. Materials and Methods

This systematic review was conducted following the preferred reporting items for systematic reviews and meta-analyses (PRISMA) guidelines [69,70,71]. The completed PRISMA checklist is provided in the Supplementary Materials (Table S5).

### 2.1. Literature Search and Selection

A systematic and comprehensive literature search was carried out to identify relevant fMRI studies published up to September 2023, and updated with new studies published until May 2025, through the databases *PubMed* (https://pubmed.ncbi.nlm.nih.gov/ (accessed on 1 May 2025)) and *Web of Science* (https://webofknowledge.com (accessed on 1 May 2025)).

The search input string was originated combining the chosen keywords with the Boolean operators AND and OR. The *PubMed* search input was: (“Inhibition AND fMRI AND Impulsivity”). The *Web of Science* search input was: TS = (“Inhibition AND fMRI AND Impulsivity”). Furthermore, some additional records were searched from the references of identified studies and published reviews (e.g., [72,73,74,75,76]).

All identified records were first screened based on their titles, then on their abstracts. Finally, the full texts of the remaining studies were carefully examined for eligibility. Studies meeting the following criteria were included: (1) experimental studies published in peer-reviewed journals; (2) studies that enrolled healthy adults (mean age range between 18 and 60)—when studies with patients included a control group, the data for healthy controls were included if the results were reported separately; (3) fMRI studies that used non-ecological human–computer tasks (Go/Nogo and/or SST as behavioral tasks, reporting data on reaction times in the Go conditions); (4) studies that assessed impulsivity traits using the self-report questionnaire BIS-11 and whose subject samples achieved a mean score ≥ 52 for the BIS-11 [77] or ≥13 for the BIS-Brief [78] suggesting relatively higher levels of trait impulsivity within non-clinical populations; and (5) studies with whole-brain GLM analysis performed on fMRI data, for which the coordinates of activation foci were reported (provided in the Montreal Neurological Institute—MNI or Talairach reference space).

Exclusion criteria were: (1) review articles, case reports, and conference proceedings; (2) studies conducted on the elderly population or children/adolescents; (3) studies employing behavioral tasks other than Go/Nogo and/or SST or using ecological settings different from human–computer ones or stimuli with emotional content or reward; (4) studies that assessed impulsivity traits using self-report questionnaires other than the BIS-11 or reported non-impulsive samples (i.e., mean scores lower than the described limits); and (5) studies conducted using other neuroimaging techniques, brain modulation, or fMRI studies that reported only results from ROI analysis.

Following these selection criteria, we created the Impulsivity Group (hereafter, IG) including participants with impulsive personality traits.

Additionally, to define a Control Group (hereafter, CG), we re-screened the database previously published in a meta-analysis conducted by our research group [19]. Importantly, this database had already been constructed through a rigorous PRISMA-guided selection procedure and included inhibitory-control fMRI studies meeting the same strict inclusion criteria. Therefore, the database was used as a pre-screened pool of eligible studies. Starting from the 68 eligible studies included in the original database, we independently re-screened and selected the records meeting both the previous and the following additional inclusion criteria: (1) studies conducted in healthy adults that assessed impulsivity traits using the self-report questionnaire BIS-11 and whose subject samples achieved a mean score lower than 52 for the BIS-11 [77] or 13 for the BIS-Brief [78]; and (2) studies reporting mean reaction times (RTs) in the Go conditions, matching as closely as possible those of the IG group. Following these criteria enabled us to select a homogeneous set of studies, ensuring more robust measures [79]. For datasets in which Go RTs were not explicitly reported, a global median RT value calculated across all studies included after the matching procedure was assigned. Importantly, this imputation was used solely to allow their inclusion in the ALE analysis while preserving the minimum number of experiments recommended for reliable ALE analyses [80] and did not influence the initial group-matching procedure.

All the authors selected and approved the inclusion criteria and the keywords for the literature search. To minimize the risk of selection bias and ensure quality of inclusion, the entire procedure was carried out in a double-check fashion by independent investigators, as recommended by recent guidelines [81]. Four of the authors independently screened all articles included (C.N., C.P., F.G., G.G.).

### 2.2. Activation Likelihood Estimation (ALE)

Coordinate-based ALE meta-analyses [80,82,83,84] implemented in GingerALE 3.0.2 software (www.brainmap.org/ale) were adopted in the present study to analyze the datasets gathered.

ALE is a coordinate-based meta-analysis that receives in input peak coordinates of functional studies. Previous methodological papers [80,85] detail the algorithm. In brief, the ALE algorithm evaluates, controlling for the sample size, the convergence of activation foci from different neuroimaging studies, modeled as probability distributions of activation [85] at given coordinates, against null distributions of random spatial associations between studies.

In our study, the statistical parameters were set as a cluster-level family-wise-error-corrected threshold of *p* < 0.01 and the cluster-forming threshold at voxel-level *p* < 0.005 against a null-distribution generated by 2000 random permutation tests [86,87] (Table S1 details the study selected). For studies that reported Talairach space, we converted the Foci coordinates reported in Talairach space into MNI space with a custom MATLAB code (Version 26.1) based on Matthew Brett’s transformation function ‘tal2mni’ (https://imaging.mrc-cbu.cam.ac.uk/imaging/MniTalairach; the code is available upon request).

In addition, conjunction analyses and contrast analyses were performed in GingerALE to compare the results obtained in the IG and CG. The parameters of contrast analyses were set as an uncorrected *p* < 0.005 with 10,000 permutations and a cluster-size threshold of 200 mm^3^ [88]. In line with previous studies, the differences in *p*-values and the number of permutations vary between within-group, conjunction, and contrast analyses to minimize the risk of Type I and Type II errors (e.g., [5,9,19,80,85,86,88,89]). Considering the inclusion criteria for both groups, the selected studies may vary in the task design used (e.g., Go/NoGo, SST tasks). To minimize potential confounding effects related to differences in task design, studies were matched according to Go RTs. Reaction times to Go stimuli, irrespective of the specific paradigm employed, are widely recognized as a robust indicator of inhibitory-control demand [7,19,39,90,91,92,93,94,95,96,97]. To assess whether potential variations in task design might introduce heterogeneity in the results, an additional sensitivity analysis restricted to studies employing the Go/NoGo task was performed. This analysis was specifically intended to evaluate whether the between-group findings were robust. The same parameters described above were adopted for both within-group and conjunction analyses (cluster-level family-wise-error-corrected threshold of *p* < 0.01; cluster-forming threshold at voxel-level *p* < 0.005; 2000 random permutation tests) as well as for contrast analysis (uncorrected *p* < 0.01 with 10,000 permutations).

Whole-brain maps of the thresholded ALE images were visualized in Mango V.4.0.1 (http://rii.uthscsa.edu/mango/), which is an anatomical image overlay program, and superimposed onto a standardized anatomical template in MNI space.

## 3. Results

### 3.1. Results of the Study Search

**Figure 2:**
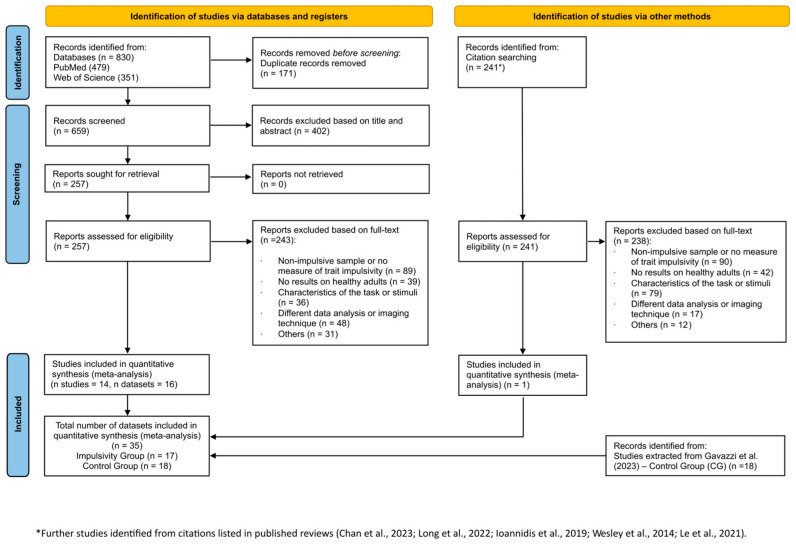
PRISMA flowchart of the literature search and selection process for the ALE meta-analysis [72,73,74,75,76].

Our literature search for the IG (i.e., studies conducted on samples with impulsive personality traits) yielded 498 potentially eligible studies (the flow chart illustrating the article selection process is shown in Figure 2). At the end of the process, we included 17 datasets gathered by 15 studies in the quantitative analysis, encompassing a total of 501 subjects and yielding 167 foci. Two studies [63,64] provided two independent eligible datasets each, which were treated as separate datasets in the analyses.

Concerning the CG (i.e., studies conducted on non-impulsive participants), out of the 68 eligible studies evaluated by [19] Gavazzi, 18 studies were selected and included in the quantitative analysis, comprising a total of 387 subjects and 241 foci.

The main characteristics of meta-analyzed studies [47,63,64,65,66,67,68,98,99,100,101,102,103,104,105,106,107,108,109,110,111,112,113,114,115,116,117,118,119,120,121,122,123] are reported in Supplementary Materials, Table S1. A group-level summary of the two IGs and CGs is shown in Table 1. A complete list of the 68 studies [8,47,63,66,67,99,100,101,102,103,104,105,106,107,108,109,110,111,112,113,114,115,116,117,121,123,124,125,126,127,128,129,130,131,132,133,134,135,136,137,138,139,140,141,142,143,144,145,146,147,148,149,150,151,152,153,154,155,156,157,158,159,160,161,162,163] included in the original database from Gavazzi et al. [19], from which the CG studies were selected, is reported in Supplementary Materials, Table S2. According to independent t-test and taking into account the Bonferroni correction of *p*-values for the considered multiple comparisons (α bonf = 0.05), the reaction times of the two groups were not statistically different: IG vs. CG (t_33_= −1.692, *p* > 0.05). To assess the potential contribution of confounding factors, we also conducted four ANOVAs weighted according to the sample size (i.e., number of participants in each study) with Groups as the independent variable and found that the two groups of studies did not statistically differ for age—F(1, 33) = 0.005; *p* = 0.944, gender—F(1, 33) = 0.08; *p* = 0.780, proportion of NoGo stimuli presented in the task—F(1, 33) = 0.008; *p* = 0.931, and TR—F(1, 33) = 2.863; *p* = 0.100. Since no consensus gold-standard method is currently available for evaluating publication bias in ALE meta-analyses, we assessed funnel plots, which did not reveal any asymmetry in either group of studies (see Supplementary Materials, Figure S1).

For the sensitivity analysis, a subset of studies was selected that employed only the Go/NoGo task for each CG and IG. Specifically, 18 datasets were included in the control group (hereafter CG-Go/NoGo), consisting of a total number of 387 subjects and 241 foci, whereas 15 datasets were considered for the IG (hereafter IG-Go/NoGo), excluding the investigations conducted with an SST [63,120] for a total number of 459 subjects and 153 foci.

### 3.2. ALE Results

**Figure 3:**
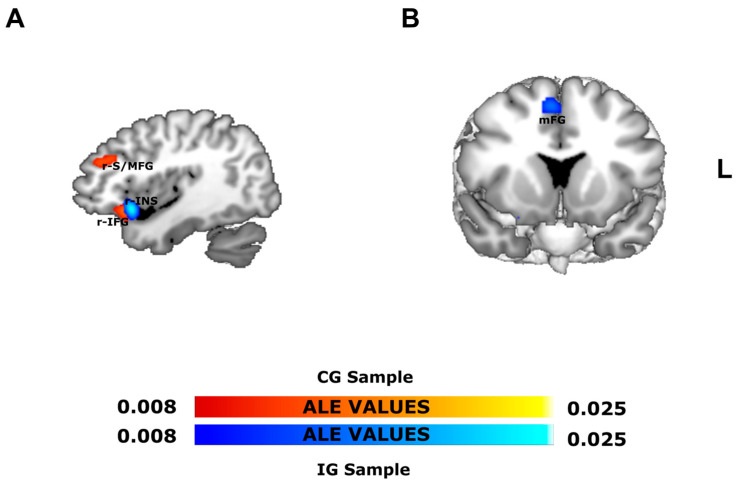
ALE meta-analysis map for the Inhibition process of our data selection in the two groups. Panel (**A**) Sagittal brain view. The algorithm converged for CG sample (in white-red) on the right Middle Frontal Gyrus and part of the Superior Frontal Gyrus (r-S/MFG), right Insula (r-Ins) and extended to r-IFG. The algorithm converged for IG sample (in white-blue) on right Insula (r-Ins) and extended to r-IFG. Panel (**B**) Coronal brain view. The algorithm converged for IG sample (in white-blue) bilaterally in Anterior Cingulate Cortex and extending to the Medial Frontal Gyrus (mFG in figure). −*p* < 0.01 cluster-level corrected inference using *p* < 0.005 uncorrected at voxel-level as the cluster-forming threshold generated by 2000 random permutation tests. Figure created with Mango V.4.0.1 (http://rii.uthscsa.edu/mango/) and Inkscape V.1.4.0.

The ALE meta-analysis of the CG (Figure 3 and Table 2) identified the largest cluster (3112 mm^3^) in the right Insula and extending in the right Inferior Frontal Gyrus (r-IFG), followed by a cluster encompassing part of the right Middle and part of the Superior Frontal Gyrus (r-MFG and r-SFG—2672 mm^3^).

The analysis of the IG (Figure 3 and Table 2) revealed the largest cluster in terms of size (4528 mm^3^) in the right Insula and r-IFG. A second cluster (2184 mm^3^) centered on the Anterior Cingulate Cortex and extending to the Medial Frontal Gyrus (ACC and mFG, respectively) was identified.

**Figure 4:**
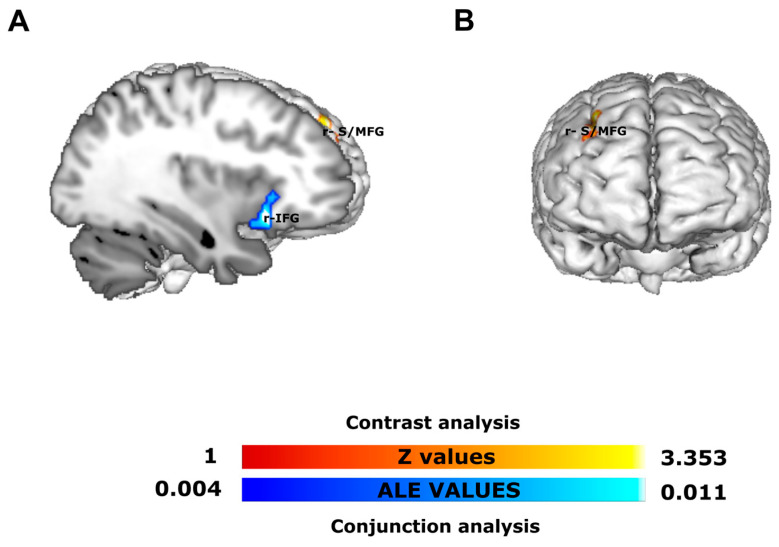
Group Contrast and Conjunction analyses. Panel (**A**) 3D sagittal brain view. The conjunction analysis (white-blue) shows a cluster (1136 mm^3^) centered at (36.5, 19.9, −6.9) in the right Insula and extending in Inferior Frontal Gyrus (r-IFG). By contrasting the IG and CG (white-red) we observed higher convergence of activity for the CG in the cluster (312 mm^3^) located in the right Middle Frontal Gyrus (r-MFG) and Superior Frontal Gyrus (r-SFG) centered at (25.8, 49.5, 37.1). Panel (**B**) 3D anterior brain view. This panel depicts a different view of the results of the contrast analysis (between the IG and CG), showing the same cluster of Panel A (white-red) located in the right Middle Frontal Gyrus (r-MFG) and Superior Frontal Gyrus (r-SFG). Statistical threshold −*p* < 0.005; 10,000 permutations; Cluster-size threshold = 200 mm^3^. Figure created with Mango V.4.0.1 (http://rii.uthscsa.edu/mango/) and Inkscape V.1.4.0.

By contrasting the CG and IG we observed higher convergence of activity for the CG (as shown in white-red in Figure 4, Table 3) in the cluster located in the right Middle and Superior Frontal Gyrus (312 mm^3^).

In the conjunction analysis of the IG and CG, we found common activation (as shown in Figure 4 and Table 3) in a cluster centered in the right Insula and extending to the Inferior Frontal Gyrus (1136 mm^3^).

#### Sensitivity Analysis Result

Concerning the sensitivity analysis, we added a meta-analysis of studies of both the CG and IG that used only the Go/NoGo task. The ALE meta-analysis of the CG- Go/NoGo subgroup (Table S2) identified the same clusters as the primary analysis for location and dimension, showing the involvement of the right Insula, the right Inferior Frontal Gyrus, part of the right Middle and Superior Frontal Gyrus. The within analysis of the IG-Go/NoGo subgroup (Table S3) reported the involvement of one cluster (4640 mm^3^) encompassing the right Insula and r-IFG; the second cluster (2288 mm^3^) lying bilaterally over the Cingulate Gyrus and the Medial Frontal Gyrus; and a third and a fourth cluster, both left lateralized located in the Caudate (1504 mm^3^) and the Precentral Gyrus (1448 mm^3^). By contrasting CG-Go/NoGo and IG-Go/NoGo subgroups, we observed higher convergence of activity for the CG-Go/NoGo (detailed in Table S4) in a cluster located in the right Middle and Superior Frontal Gyrus (368 mm^3^). Importantly, the cluster identified in the CG-Go/NoGo > IG-Go/NoGo contrast spatially overlapped the one observed in the primary CG > IG comparison, involving the right Middle and Superior Frontal Gyrus. These findings therefore replicate the between-group effect within a more homogeneous subset of studies employing the same inhibitory-control paradigm. In the conjunction analysis of the IG-Go/NoGo and CG-Go/NoGo, we found common activation (Table S4) in one cluster centered in the right Insula and extending to the Inferior Frontal Gyrus (1144 mm^3^). Overall, the marked convergence between the primary analysis and the Go/NoGo-only sensitivity analysis strongly suggests that the observed between-group differences cannot be attributed to task composition. Rather, these findings appear to support the robustness and stability of the altered recruitment pattern involving higher-order right prefrontal regions in impulsive individuals.

## 4. Discussion

The aim of this meta-analysis is to explore the neural correlates of inhibitory control, with a particular emphasis on the impulsive personality trait. Our specific focus was to better explain this process by carefully comparing studies based on reaction time outcomes, exemplified by previous studies [7,19,37,90,91,92,93,97,164,165]. By employing this method, we sought to overcome any potential discrepancies in the average load of inhibitory control observed across the included studies. In particular, this approach provides the opportunity to disentangle the impact of impulsivity on performance during non-ecological inhibitory tasks employing highly simplified stimuli devoid of ecological or salient content (e.g., faces or complex visual images). This focus is supported by a recent meta-analysis showing consistent hypoactivation of prefrontal control regions, particularly the right middle frontal gyrus, across conditions characterized by impulsivity, like depression and substance use disorder [166].

The main results raised from the conjunction and the contrast analyses need to be interpreted together. Contrasting CG and IG, we observed a greater recruitment of clusters located in the right Middle and Superior Frontal Gyrus in the CG, whereas the lower portion of the prefrontal cortex and the right Insula were commonly activated both in the IG and CG. This right lateralization is in line with several evidence showing a peculiar involvement of the right hemisphere to achieve a successful inhibitory control (e.g., [11,12,13,127]).

In particular, the lower regions of the prefrontal cortex were found to be active in both groups while the upper regions were observed exclusively in the contrast analysis (CG vs. IG), suggesting a possible association between these regions and inhibitory-control differences related to impulsivity.

In line with this, recent evidence indicates greater activity in the superior regions of the prefrontal cortex in the presence of increased inhibitory demand. This implies that areas beyond the Inferior Frontal Gyrus may be crucial for preserving proper inhibition. Supporting this view, studies by [20] Apsvalka and [19] Gavazzi highlight the involvement of the Middle and Superior Frontal Gyrus in enhancing inhibitory capacity, while the Inferior Frontal Gyrus is primarily engaged during proactive phases of inhibition [21,128], in conjunction with the *salience network* [24]. These results can be interpreted in the frame of a recent explanatory model [19], which indicates that shorter reaction times reflect greater inhibitory demand, recruiting a larger portion of the upper right prefrontal cortex. In agreement with this model and with a wide body of literature, confirmatory analyses within the examined CG and IG groups initially showed consistent activation in brain regions commonly associated with inhibitory control [28,37,89,117,167,168,169,170,171], specifically the Anterior Cingulate Cortex, Medial Frontal Gyrus and right prefrontal cortex (see Figure 1).

Therefore, according to the framework of the Proactive–Reactive Model [19], and consistent with other independent supportive studies [13,21], the comparison between the two groups (CG vs. IG) may suggest that impulsive individuals predominantly exhibit alterations in reactive control components. This pattern may suggest a reduced engagement of the higher regions of the prefrontal cortex, such as r-S/MFG, which could be reflected in the lower ability to maintain effective inhibition under more demanding conditions—a characteristic trait of impulsive individuals [55,133,172]. This reduced recruitment of upper right prefrontal regions may reflect altered functional organization and network embedding in impulsive individuals and aligns with recent evidences indicating reduced engagement of right prefrontal control regions as a core neural feature of impulsivity [166,167,173]. This perspective may help to elucidate how individuals with impulsivity traits adapt to environmental requirements, especially in non-ecological ones.

In a recent meta-analysis [174], Gell and colleagues, combining behavioral inhibitory failures as an indirect measure of impulsivity with serotonergic neurotransmitter concentrations, have identified three potential networks for inhibition: frontoparietal, temporoparietal and cingulo-Insular systems. As a matter of fact, we know that frontoparietal regions (pre-SMA, MFG) are part of the dorsal attentional network that allows a top-down control of the visuo-spatial attentional orientation [24] towards relevant stimuli. At variance, cingulate–insular regions such as the insula and the Anterior Cingulate Cortex are involved in the *salience network* [24,175,176]. These regions allow the initiation of motor control and the switching between higher-order systems, such as transitioning from Proactive to Reactive Inhibitory Control, by detecting salient stimuli like a stop signal [5,19]. These pieces of evidence, if combined, might corroborate the functional results we observed, adding that, at the neurochemical level, the ineffective communication between these brain regions may underlie the behavioral dysfunction due to the impulsive trait.

An additional sensitivity analysis, focusing exclusively on studies employing the Go/NoGo task, was performed within the CG-Go/NoGo and IG-Go/NoGo subgroups to assess the influence of different task designs. The contrast of CG-Go/NoGo versus IG- Go/NoGo in this sensitivity analysis corroborated the involvement of the Middle and Superior Frontal Gyrus in the CG- Go/NoGo group. In fact, the same activation foci observed in the main analysis were replicated. These outcomes suggest that differences associated with impulsivity in inhibitory responses are minimally influenced by task design, thereby reinforcing the robustness of our main analysis. Importantly, the replication of the main CG > IG effect within the Go/NoGo-only subgroup indicates that the difference observed in the right middle/superior frontal regions in impulsive individuals cannot be explained by the inclusion of SST paradigms in the primary analysis. This strengthens the interpretation that altered engagement of higher-order right prefrontal regions may be associated with trait impulsivity during inhibitory-control processing.

The literature emphasizes that the Go/NoGo task predominantly engages Proactive Inhibitory Control [177,178,179,180]. In line with this, our sensitivity analysis suggests greater involvement of brain regions in the left hemisphere in individuals with a high trait of impulsivity in the within-group analysis. This pattern may reflect the greater proactive demand associated with the Go/NoGo task design. In particular, the additional involvement of the left caudate may reflect greater recruitment of cortico-striatal circuits associated with motor-response selection and inhibitory preparation, whereas activation of the left precentral gyrus could be related to compensatory motor-planning processes during response suppression. Recent evidence suggests that increased inhibitory demands or higher levels of impulsivity may recruit additional left-hemisphere support when right-lateralized control systems are inefficient, through a hierarchical upward shift in frontal engagement from inferior to middle and ultimately superior frontal regions [19,181,182,183]. Nevertheless, since these findings emerged only in the within-group sensitivity analysis and did not survive the between-group contrast, they should be interpreted cautiously, although they may still present a pattern of left hemisphere recruitment in individuals with impulsive traits that does not appear to characterize subjects without impulsive traits.

Coherently, [61] Pan reported a negative correlation between impulsivity measures (e.g., BIS-11, UPPS-P) and the volume of various prefrontal cortex regions involved in the inhibitory process, as the right Superior and Middle Frontal Gyrus and the Middle Anterior Cingulate Cortex. Conversely, positive correlations were identified between impulsivity traits and the volume of the right Superior Temporal Gyrus, the Inferior Frontal Gyrus, and the left Postcentral Gyrus.

The main result of the present meta-analysis highlights the involvement of both the superior and middle frontal gyrus to exhibit a successful inhibition when comparing the control group with the trait-impulsive population. Thus, our work supports the notion that impulsivity may affect the reactive circuitry of inhibitory control [4,47,55,58,117]. One possible interpretation is that impulsive individuals may rely more on the proactive than the reactive phase of cognitive control to successfully inhibit their impulses. This may suggest that, in impulsive individuals, a potential mechanism of inhibitory control may be driven more by the proactive process to counterbalance and compensate for alterations in reactive control mechanisms. However, this hypothesis warrants further investigation, as it cannot be directly tested within the present ALE framework.

This finding is particularly noteworthy, as cognitive control deterioration is commonly observed in the elderly. A deeper understanding of the processes and brain regions involved in proactive and reactive control could help develop compensatory training programs in older adults and/or impulsive populations.

Moreover, such insights are increasingly relevant in modern environments characterized by pervasive interactions with technological devices. These contexts require constant monitoring and regulation of automatic responses, placing additional demands on inhibitory control. Our results suggest that altered recruitment of right prefrontal regions in impulsive individuals may help understand vulnerability to maladaptive behaviors in these settings and could guide the development of adaptive artificial systems that support decision-making.

We acknowledge some limitations. First of all, to obtain a homogeneous comparison between studies analyzing volumes associated with a similar behavioral performance, we had to consistently reduce the number of selected studies applying very stringent selection criteria. In addition, although the number of included experiments met the minimum recommendations for ALE analyses [80], the relatively limited number of datasets and the lower number of activation foci in the IG group might have reduced the statistical sensitivity of between-group contrasts, particularly with respect to the IG > CG contrast. Additionally, the CG > IG contrast revealed a limited size of the right SFG/MFG cluster, suggesting that this finding should be interpreted cautiously and confirmed by future studies. Another flaw of this study concerns the poor temporal resolution of the fMRI technique of neuroimaging that, while it allows us to investigate the spatial component of the network of inhibitory control in depth, can limit us in resolving the inherent temporal factors of the phenomenon. Notably, our metanalysis based on foci of activations gathered from impulsivity studies differed from the results obtained in morphometrical metanalyses. In fact, morphometrical metanalyses showed more pronounced differences in the left prefrontal cortex that here are observed exclusively in the within-level of the sensitive analysis [50,54,57,59,60,61,167,184,185]. In light of the limitations outlined above, future meta-analyses will be necessary to further validate and replicate the present findings.

## 5. Conclusions

In conclusion, contrasting the CG and IG through meticulous matching based on reaction times, we revealed distinct activation patterns in the prefrontal cortex. Both groups showed activation in the lower prefrontal cortex and the right Insula. However, only impulsive individuals did not exhibit significant activity in upper prefrontal cortex regions. Our results may be consistent with altered recruitment of brain regions commonly associated with reactive components of inhibitory control, characterized by reduced engagement of higher prefrontal cortex regions such as r-S/MFG during inhibition.

The present work was conceived as a tool to further clarify the spatial dynamics underlying inhibitory processes. Nevertheless, a more comprehensive understanding requires additional information on the temporal dynamics of such a phenomenon.

## Figures and Tables

**Table 1:** Characteristics of the studies included at the group level. The table summarizes the characteristics of the datasets included in the coordinate-based meta-analysis for the control group (CG) and the impulsive group (IG). For each group, the number of datasets, total sample size, and number of activation foci are reported, together with participants’ mean age, gender distribution (female/total ratio), percentage of NoGo trials (% NoGo), and mean reaction time during Go trials (RT_Go, in milliseconds). Values are presented as mean (standard deviation), unless otherwise specified.

Group-Level Studies Characteristic							
Group	Group size (N)	Sample size (N)	Foci (N)	Age	Gender	% NoGo	RT_Go (ms)
CG	18	387	241	29.764 (4.896)	0.52	0.312 (0.183)	342.26 (18.346)
IG	17	501	167	29.619 (7.024)	0.491	0.307 (0.137)	365.8 (55.972)

**Table 2:** Results from ALE meta-analysis of both CG and IG samples. From left to right, the table reports the number of clusters, stereotaxic MNI coordinates of local maxima, ALE scores, *p* scores and anatomical labeling (with corresponding Brodmann area) of the clusters that were consistently associated with successful inhibition of both the CG and IG. −*p* < 0.01 cluster-level corrected inference using *p* < 0.005 uncorrected at voxel-level as the cluster-forming threshold generated by 2000 random permutation tests.

Results from ALE Meta-Analysis of Both CG and IG Samples. MNI Coordinates. BA = Brodmann’s Area.						
CG sample: ALE metanalysis computed from our study selection						
Cluster	x	y	z	ALE	*p*	Label (Nearest Gray Matter within 5 mm)
1	32	24	−6	0.024634913	2.81 × 10^−8^	Right Claustrum
	44	30	−10	0.016408809	1.68 × 10^−5^	Right Inferior Frontal Gyrus.BA 47
	32	14	−10	0.008899848	0.002954644	Right Claustrum
2	36	40	26	0.015126572	4.42 × 10^−5^	Right Superior Frontal Gyrus.BA 9
	36	50	26	0.014915976	5.20 × 10^−5^	Right Superior Frontal Gyrus.BA 9
	42	34	26	0.014061867	1.00 × 10^−4^	Right Middle Frontal Gyrus.BA 9
	28	52	42	0.009665801	0.001798898	Right Superior Frontal Gyrus.BA 8
	20	52	32	0.008833956	0.003086074	Right Superior Frontal Gyrus.BA 8
	26	50	32	0.008613995	0.003581225	Right Superior Frontal Gyrus.BA 9
IG sample: ALE metanalysis computed from our study selection						
Cluster	x	y	z	ALE	*p*	Label (Nearest Gray Matter within 5 mm)
1	38	18	−8	0.021293892	3.88 × 10^−7^	Right Insula.BA 13
	30	22	4	0.01169453	3.76 × 10^−4^	Right Claustrum
	52	16	−4	0.010143784	9.16 × 10^−4^	Right Insula.BA 13
	18	16	−8	0.009831958	0.001113312	Right Putamen
2	−4	30	20	0.01622326	1.63 × 10^−5^	Left Anterior Cingulate.BA 24
	6	10	50	0.015761267	2.36 × 10^−5^	Right Medial Frontal Gyrus.BA 6
	0	20	40	0.013731107	1.05 × 10^−4^	Left Cingulate Gyrus.BA 32
	−4	26	30	0.010306846	8.32 × 10^−4^	Left Cingulate Gyrus.BA 32
	−6	12	42	0.008577172	0.002783306	Left Cingulate Gyrus.BA 32

**Table 3:** Results from ALE meta-analysis of both CG—IG samples contrast and CG and IG samples conjunction. From left to right, the table reports the number of clusters, stereotaxic MNI coordinates of local maxima, ALE scores, *p* scores, and anatomical labeling (with corresponding Brodmann area) of the clusters that were consistently associated with successful inhibition of both conjunction and contrast analysis. The parameters of contrast analyses were set as an uncorrected *p* < 0.005 with 10,000 permutations and a cluster-size threshold = 200 mm^3^.

Results from ALE Meta-Analysis of Both CG—IG Samples Contrast and CG & IG Samples Conjunction. MNI Coordinates. BA = Brodmann’s Area.					
Contrast CG—IG: ALE meta-analysis computed from our study selection					
Cluster	x	y	z	*p*	Label (Nearest Gray Matter within 5 mm)
1	25	50	36	3.00 × 10^−4^	Right Superior Frontal Gyrus.BA 8
	28	51	42	5.00 × 10^−4^	Right Superior Frontal Gyrus.BA 8
Conjunction IG & CG samples: ALE meta-analysis computed from our study selection					
Cluster	x	y	z	ALE	Label (Nearest Gray Matter within 5 mm)
1	38	20	−10	0.014739955	Right Inferior Frontal Gyrus.BA 47
	32	24	2	0.010000105	Right Claustrum
	32	14	−10	0.008899848	Right Claustrum

## Data Availability

No new data were created or analyzed in this study. Data sharing is not applicable to this article.

## Supplementary Materials

The following supporting information can be downloaded at: https://www.mdpi.com/article/10.3390/brainsci16060609/s1. Figure S1: Funnel plots for publication bias assessment; Table S1: Characteristics of study selected for CG and IG samples; Table S2: Characteristics of the original sample of studies from Gavazzi et al. (2023); Table S3: Results from ALE meta-analysis of both CG-Go/NoGo and IG-Go/NoGo subsamples; Table S4: Results from ALE meta-analysis of both CG-Go/NoGo–IG-Go/NoGo and IG-Go/NoGo–CG-Go/NoGo samples contrast, and CG-Go/NoGo and IG-Go/NoGo samples conjunction. Table S5: PRISMA 2020 Checklist.
