# Supplementary material for: Neural Correlates of Inhibitory Control in Impulsivity Traits in Non-Ecological Human–Computer Tasks: An ALE Meta-Analysis
Source: Brain Sci. 2026 Jun 3;16(6):609. doi: 10.3390/brainsci16060609 (PMC13296890; doi:10.3390/brainsci16060609)
Supplement: Supplementary file 1 [file brainsci-16-00609-s001.zip › Supplementary Material.pdf]

Supplementary Material

Table S1. Characteristics of study selected for CG and IG samples. Characteristics of studies included in the quantitative analysis (the first part of the table refers to the descriptives of Control Group studies selected, whereas the second part refers to the Impulsive Group studies selected). From left to right, the table reports the progressive study number (N), the authors and publication year of each study, the number of subjects, their age (in years) and the female/male ratio, the behavioral performance at the task (Go reaction times, Go RTs). Finally, the original contrasts included in each ALE meta-analysis are reported, together with the corresponding number of foci, the standard anatomical space, and the description of the task employed. Finally, the BIS-11 or the BIS-Brief mean score is reported. The asterisk (*) indicates that the values were re-computed by the authors from those provided in the selected studies to obtain unique average values. Superscript "a" indicates imputed Go RT values. Specifically, when Go RTs were not reported (4 datasets of the 35 analyzed), the median of the average reaction times calculated across all 35 datasets (both CG and IG groups) was used.

Table S1. Characteristics of study selected for CG and IG samples.

N	Ref	Author (year)	Sample (N)	Age (years)	f/m (ratio)	Go RT (ms)	Contrasts	Foci	Space	fMRI task	Impulsivity (BIS-11 / BIS-Brief)
1	[99]	Chikazoe et al. (2008)	25	range 20-27 y.o.	15/10	288,7* ms	Nogo/Stop vs Go	52	Tal	Go/Nogo task with three types of trial are intermixed in pseudorandom order: in the frequent-go and infrequent-go trials, no-go trial. For each trial, a colored circle was presented for 400 ms, which was followed by a 400-ms intertrial interval. In total, 1440 (75.4%) frequent-go, 234 (12.3%) infrequent-go, and 234 (12.3%) no-go trials were intermixed in pseudorandom order.	–

2	[100]	Watanabe et al. (2002)	11	mean 25 y.o.	2/9	315 ms	Nogo/Stop	5	Tal	Go/Nogo task. Presentation of a preparation cue for 2 s (green square). Then a GO or NO-GO/Stop signal (a red or blue square) was randomly chosen and presented for 1 s. The subjects were instructed to press a mouse button immediately after the GO signal was presented, and not to move when a NO-GO signal was presented. Random series of 30 GO and 30 NO-GO trials.	–
3	[101]	McNab et al. (2008)	11	mean 24 y.o.	7/4	329 ms	Nogo/Stop vs Go	6	MNI	Go/No-Go task: 50% of trials involved the presentation of a yellow square for 1300 ms (go trials) and participants were required to press a button. For 25% of the trials a yellow triangle indicated that participants should not make a response (Stop/No/go trials). For the remaining 25% of trials the stimulus consisted of a blue square, which also required a button press.	–
4	[102]	Liddle et al. (2001)	16	mean 30.2 y.o.	7/9	338 ms	Nogo/Stop vs Baseline	15	Tal	Go/Nogo task. The presentation of each trial began with a descending series of asterisks. Participants were instructed to respond with their right index finger every time ’X’ was presented (50%) and not to respond when ’A’ was presented (50%).	–

										Each stimulus was presented for a period of 250 ms.	
5	[103]	Garavan et al. (2002)	14	mean 30 y.o.	10/4	338 ms	Nogo/Stop vs Baseline	16	Tal	Go/Nogo task. Letters X and Y were presented serially in an alternating pattern and subjects were required to make a button press response to each letter. Responses were to be withheld to lure stimuli: a lure occurred when the alternation was interrupted (e.g., the fifth stimulus in the train X-Y-X-Y-Y-X-Y). Subjects completed four runs that contained 1180 targets (GO stimuli) and 80 lures (NOGO stimuli).	–
6	[104]	Garavan et al. (2003)	16	mean 31 y.o.	10/6	339* ms	Nogo/Stop vs Baseline	7	Tal	Go/Nogo task. Subjects were presented with a 1Hz serial stream of alternating letters, X and Y, and were required to make a button press response to each letter except when the alternation order was broken (e.g., subjects would respond to each letter except the fifth in the following sequence: X, Y, X, Y, Y, X). There were 448 GOand52NOGOst imulipresented in each conflict condition resulting in an average interval between	–

										the NOGO stimuli of 9.6 s.	
7	[105]	Mostofsky et al. (2003)	48	mean 27.45 y.o.	24/24	339 ms	Nogo/Stop	3	Tal	Go/Nogo task. Cues consisted of drawings of green (Go-82%) and red (No-go) spaceships. Subjects were instructed to push the button in response to green spaceships only and not to push during No-go signal.	–
8	[106]	Zheng et al. (2008)	18	range 22-40 y.o.	10/8	340,8 ms	Nogo/Stop vs Go	8	Tal	Go/Nogo task. n a go trial, the ''+'' turned into a circle after a randomized time interval (between 1500 and 2500 msec). Subjects were instructed to press a button with their right index finger as soon as they saw the go signal. On a stop trial, a stop signal (an ''x'') appeared and subjects were instructed not to press the button. The task consisted of 75% go trials and 25% stop/no-go trials.	–
9	[107]	Yanaka et al. (2010)	27	mean 23.45* y.o.	13/14	342,15* ms	Nogo/Stop vs Go	5	MNI	Go/Nogo task. The subjects were initially presented with a central fixation cross. After a relatively long ITI of 12–14 s, the color of the fixation cross changed from white to yellow as a warning stimulus. Following a variable time period (2–6 s), a blue or red square	–

										was presented as the Go signal or NoGo signal, respectively. When the Go signal was presented, the subjects had to respond by pressing a button with their right thumb as quickly as possible. The AN task was identical to the AW task including 40 Go and 40 NoGo trials.	
10	[108]	Kaladjian et al. (2007)	21	mean 35.7 y.o.	2/19	343,7 ms	Nogo/Stop vs Go	11	Tal	Go/No-go task. After a series of cues which lasted 5 s, either the Go stimulus (letter 'X') or the NoGo stimulus (letter 'A') was presented for 250 ms. Participants were asked to respond to each presentation of the Go stimulus, by pressing a button, and to withhold response to each presentation of the NoGo stimulus. Each run contained 25 NoGo and 25 Go trials, presented in a pseudo-random order.	–
11	[109]	Criaud et al. (2017)	20	range 20-42 y.o.	10/10	344,5* ms	Nogo/Stop vs Go	13	MNI	Go/Nogo task. Participants were asked to react to visual go stimuli by pressing a button with the right thumb. At the beginning of a trial, participants saw a fixation point that could be red (main condition: indicated that either a go stimulus, a no-go	–

										stimulus or no stimulus at all could occur) or green (control condition). Each session consisted of 20 go trials, 20 no-go trials, 20 go_control trials and 20 catch trials (no stimulus), randomly presented.	
12	[110]	Kelly et al. (2004)	15	mean 30 y.o.	10/5	346,9* ms	Nogo/Stop vs Go	23	Tal	Go/Nogo task. The letters X and Y were presented serially in an alternating pattern and subjects were required to make a button press to each letter. Subjects were instructed to withhold a response to NOGO stimuli: an interruption to the alternating pattern whereby a letter was presented twice in a row. Subjects completed four runs comprising 1152 GOs and 100 NOGOs.	–
13	[111]	Hester et al. (2004)	15	mean 30 y.o.	10/5	355* ms	Nogo/Stop vs Go	21	Tal	Go/Nogo task. Subjects were required to make a button press response to each letter (X or Y). Responses were to be withheld to no-go events, which on 50% of occasions was preceded by a visual cue occurring two to seven trials in advance of the no-go event. A lure occurred when the alternation was interrupted (e.g., the fifth stimulus in the train X–Y–	–

										X–Y–Y–X–Y). Four runs that contained 1176 targets (go stimuli) and 80 lures (no-go stimuli) that included an equal distribution of cued and uncued lures.	
14	[112]	Kaladjian et al. (2009)	10	mean 41.5 y.o.	5/5	355,35 ms	Nogo/Stop vs Go	12	Tal	Go/Nogo task. Subjects had to respond pressing a button as quickly as possible to Go trials (letter 'X') and to withhold response to NoGo trials (letter 'A'). Each run contained 25 NoGo and 25 Go trials, presented in a pseudo-random order.	_
15	[113]	Kaladjian et al. (2009)	20	mean 34.6 y.o.	10/10	356 ms	Nogo/Stop vs Go	16	Tal	Go/Nogo task. Subjects had to respond pressing a button as quickly as possible to Go trials (letter 'X') and to withhold response to NoGo trials (letter 'A'). Each run contained 25 NoGo and 25 Go trials, presented in a pseudo-random order.	_
16	[114]	Mazzola-Pomietto et al. (2009)	16	mean 34.6 y.o.	10/6	360 ms	Nogo/Stop vs Go	7	Tal	Go/Nogo task. Subjects had to respond pressing a button as quickly as possible to Go trials (letter 'X') and to withhold response to NoGo trials (letter 'A'). Each run contained 25 NoGo and 25 Go trials, presented in a pseudo-random order.	_

17	[115]	Pornpattananangkul et al. (2016)	58	mean 23.25* y.o.	29/29	363,98* ms	Nogo/Stop vs Go	10	MNI	Go/Nogo task. Participants were asked to respond with their right index finger to every letter except for the designated "nontarget" ("v" and "レ"). In the Go/No-Go condition were presented 10 targets and 10 nontargets. Each of 20 letters in each block was presented for 500 ms, followed by an inter-stimulus interval of 1 s, giving rise to a 30-s block.	–
18	[116]	Gavazzi et al. (2017)	26	mean 29.5 y.o.	15/11	364,8 ms	Nogo/Stop vs Baseline	11	MNI	Go/Nogo task. Subjects were asked to press a button with their right index finger when a Go stimulus was presented. A descending series of asterisks was presented at the beginning of each trial until a single asterisk was displayed. Depending on the condition was displayed the Go ('X') or the Nogo ('A') stimulus for a temporal interval of 250 ms. The experimental session was composed by a total of 48 trials: 24 Go and 24 Nogo trials.	–

N	Ref	Author	Sample (N)	Age (years)	f/m (ratio)	Go RT (ms)	Contrasts	Foci	Space	fMRI task	Impulsivity (BIS-11 / BIS-Brief)

1	[117]	Gavazzi et al. (2018)	36	mean 30.75 y.o.	21/15	365 ms	Nogo/Stop vs Baseline	20	MNI	Go/NoGo task. Each trial started with a descending series of asterisks until a single asterisk was displayed for 250 ms and followed by a blank screen for 750 ms. Depending on the condition, the Go ("X") or the Nogo ("A") stimulus was displayed for a temporal interval of 250 ms. Subjects had to press a button as rapidly as possible after the presentation of the Go stimulus and to not answer to the Nogo stimulus	BIS-11 =71
2	[65]	Hsu et al. (2017)	20	mean 23.75 y.o.	20/0	347[a] ms	Nogo/Stop vs Go	8	MNI	Go/Nogo task. Each section consisted of 180 trials (150 go trials and 30 no-go trials) shown as numbers 1 to 9 with 200-ms durations. Participants were instructed to press the button as quickly as possible for all numbers (1, 3—9) except for the number 2. percentage of Go trial =83.3%.	BIS-11 = 69.1
3	[66]	Ko et al. (2014)	23	mean 24.35 y.o.	0/23	334 ms	Nogo/Stop	1	MNI	Go/Nogo task. Each section consisted of 180 trials (150 go trials and 30 no-go trials) shown as numbers 1 to 9 with 200-ms durations. Participants were instructed to press the button as quickly as possible for all numbers (1, 3—9) except for the number 2. percentage of Go trial =83.3%.	BIS-11 = 62.27

4	[118]	De Vito et al. (2013)	59	mean 31.6* y.o	35/24*	383,92* ms	Nogo/Stop vs Baseline	19	MNI	Go/Nogo task. Participants were asked to press a button for Go ('X', 85% of trials) and withhold responding to No-Go ('K', 15% of trials) stimuli.	BIS-11 = 57.36*
5	[47]	Asahi et al. (2004)	17	mean 25.1 y.o	7/10	325,8 ms	Nogo/Stop	11	Tal	Go/No-go task. The task consisted of eight alternating 36-s epochs of Go and No-Go conditions.Subjects viewed a series of letters once every 1500 ms and responded with a key press using the forefinger of the right hand to every letter except the letter 'X', to which they were instructed to withhold response. In the Go (control) condition, subjects were presented a random sequence of letters other than the letter 'X'. In the No-Go condition, subjects were presented with the letter 'X' 50% of the time, thus requiring a response to half the trials (Go trials) and a response inhibition to the other half (No-Go trials).	BIS-11 = 68.9

6	[67]	Chen et al. (2015)	15	mean 24.47 y.o.	0/15	360 ms	Nogo/Stop vs Go	7	MNI	Go/Nogo task. In the Go condition, a numeral from 1 to 5 was shown 20 times in a white font on a black background. In the Nogo condition, a non- target (number 0) was shown 10 times, and a target (a number from 1 to 5) was shown 10 times in a pseudorandom sequence. The participants were told to press the button as quickly as possible for all numbers except for the number 0.	BIS-11 = 62
7	[63]	van Eijk et al. (2015)	18	mean 25.28 y.o.	18/0	384 ms	Nogo/Stop vs Go	5	MNI	Go/Nogo Task. A stream of consonants was presented serially in the center of the screen. Every stimulus was displayed for 500 ms immediately followed by a blank screen for 500 ms. Subjects were instructed to make a right index finger button press for every letter (go stimulus) except for the letter "X" (nogo stimulus). Per run, 300 stimuli were presented and participants performed two runs of the Go/nogo task. Mean probability for nogo stimuli was 29% and a nogo stimulus was always followed by a go stimulus.	BIS-11 = 55.92

8	[63]	van Eijk et al. (2015)	18	mean 25.28 y.o.	18/0	493 ms	Nogo/Stop vs Go	7	MNI	SST: Each trial of the Stopsignal task started with a white fixation ring in the center of the screen. After 500 ms, a white arrow appeared within the fixation ring and the subjects were instructed to respond corresponding to the pointing direction of the arrow (go condition). In 25% of the trials the fixation ring changed color after a variable delay time (stopsignal delay, SSD) and subjects were instructed to attempt to cancel the reaction (stop condition).	BIS-11 = 55.92
9	[64]	Brown et al. (2006)	58	mean 45.3 y.o.	37/21	347[a] ms	Nogo/Stop vs Go	5	MNI	Go/Nogo task. Subjects were instructed to respond, by a button press using their right index finger, to any letter except V. There were two conditions: Block A, the go condition, in which all 20 letters in the block were targets (i.e., letters other than V), and Block B, the no-go condition, in which half the letters were targets and half were nontargets (i.e., the letter V).	BIS-11 = 57.85

10	[64]	Brown et al. (2006)	58	mean 45.3 y.o.	37/21	347[a] ms	Nogo/Stop vs Go	3	MNI	Go/Nogo task. Subjects were instructed to respond, by a button press using their right index finger, to any letter except V. There were two conditions: Block A, the go condition, in which all 20 letters in the block were targets (i.e., letters other than V), and Block B, the no-go condition, in which half the letters were targets and half were nontargets (i.e., the letter V).	BIS-11 = 57.85
11	[119]	Passamonti et al. (2008)	35	range 20-44 y.o.	0/35	302,3* ms	Nogo/Stop vs Go	16	MNI	Go/NoGo Task. Four 28-s "Go" and four 28-s "Go/NoGo" conditions were randomly presented in an interleaved design and alternating with 28-s "fixation cross" conditions as a passive rest. In the Go/NoGo condition, subjects were instructed to respond, pressing a button with their right thumb, to any letters except "V". Fifty percent of the events were targets (i.e., letters other than "V"), and the other 50% were inhibitory stimuli ("V" letters).	BIS-11 = range: 58.4 - 65

12	[68]	Passamonti et al. (2006)	24	mean 30.25 y.o.	0/24	292,795* ms	Nogo/Stop vs Go	9	Tal	Go/NoGo Task. Four 28-s “Go” and four 28-s “Go/NoGo” conditions were randomly presented in an interleaved design and alternating with 28-s “fixation cross” conditions as a passive rest. In the Go/NoGo condition, subjects were instructed to respond, pressing a button with their right thumb, to any letters except “V”. Fifty percent of the events were targets (i.e., letters other than “V”), and the other 50% were inhibitory stimuli (“V” letters).	BIS-11 = 60*
13	[120]	Filbey et al. (2013)	24	mean 24.8 y.o.	9/21	506,1 ms	Nogo/Stop vs Baseline	7	MNI	SST Task. Each trial began with a fixation circle followed by an arrow to which participants responded by button press depending on the direction of the arrow. In the Go trials, participants were given 1 s to make a left or right button press. In the Stop trials, which were indicated by a tone, participants were withheld from button pressing. The tone (i.e. StopSignal) was presented after a random delay and the duration of the blank screen was calculated at 1 s minus the response time plus the random delay. The ex-perimental	BIS-Brief = 13.8

										session was composed of a total of 48 trials (24 Go and 24 Nogo) randomized among conditions.	
14	[69]	Lee et al. (2015)	17	mean 37.9 y.o.	3/14	355,8 ms	Nogo/Stop vs Baseline	9	Tal	Go/NoGo Task. Each trial consisted of an event of 8 s: the presentation of each trial started with a descending series of numbers to build-up preparedness to respond (“5” for 250 ms followed by 750 ms blank screen, “4” for 250 ms followed by 750 ms blank screen…). Then, either a ‘go’ signal “X” or a ‘no-go’ signal “A” was presented for 250 ms followed by an additional 2750 ms black screen. 71 go trials, 71 no-go trials, and 68 resting trials in an equi-probable go and no-go task	BIS-11 = 67
15	[121]	Chen et al. (2015)	25	mean 25.64 y.o.	0/25	370 ms	Nogo/Stop vs Go	7	MNI	Go/NoGo Task. Each section consisted of 180 trials (150 Go trials and 30 No-go trials) shown as numbers 1e9 with 200-ms durations and 1425-ms interstimulus intervals. The participants were asked to press the button as quickly as possible for all numbers (1, 3e9) except for number 2. The correct performance in response to No-go stimuli was the withholding of a finger press.	BIS-11 = 65.16

16	[122]	Soloff et al. (2017)	25	mean 24.5 y.o.	25/0	347[a] ms	Nogo/Stop vs Go	8	MNI	Go/NoGo Task. Subjects were instructed to make a response only if a presented face was consistent with the instructed affective context (67% targets).	BIS-11 = 72
17	[123]	Skunde et al. (2016)	29	mean 27.25 y.o.	29/0	357,88 ms	Nogo/Stop vs Go	25	MNI	Go/NoGo Task. Participants were instructed to respond to the frequent target stimulus (square in the general no-go task) but to inhibit any reaction to the rare nontarget stimulus (circle in the general no-go task). In both modalities, each block consisted of 40 stimuli presented in a pseudorandom order with no-go stimuli occurring during 20% of the trials	BIS-11 = 58.48

Table S2. Characteristics of the original sample of studies from Gavazzi et al (2023). Sample characteristics of the studies included in the quantitative analysis, from which the datasets for the control group (CG) were selected. From left to right, the table reports the DOI; the authors and publication year of each study; the number of participants; their age (in years) and female-to-male ratio; and behavioral task performance (Go reaction times). Finally, the original contrasts included in each ALE meta-analysis are reported, together with the corresponding number of foci and a description of the task used. Adapted from Gavazzi et al. (2023)

DOI	Author (Year)	Sample	Age	f/m	Go RT	Contrast	Foci	fMRI task
https://doi.org/10.1093/cercor/bhn065	Chikazoe et al. (2008)	25	range 20-27 y.o.	15/10	288 ms	Nogo/Stop vs Go	52	Three types of trial are intermixed in pseudorandom order: in the frequent-go and infrequent-go trials, the subjects were required to press a button, and in the no-go trial, the subjects were required not to press a button. For each trial, a colored circle was presented for 400 ms, which was followed by a 400-ms intertrial interval.The relationship between color (blue/yellow) and trial type (no-go/infrequent-go) was counterbalanced across subjects.
https://doi.org/10.1006/nimg.2002.1198	Watanabe et al. (2002)	13	range 19-40 y.o.	2/11	315 ms	Nogo/Stop	5	A session consisted of 60 trials. The duration of a trial was 12 s. In each trial, a fixation point was presented for 9 s, followed by the presentation of a preparation cue for 2 s (green square). Then a GO or NO-GO/Stop signal (a red or blue square) was randomly chosen and presented for 1 s. The subjects were instructed to press a mouse button immediately after the GO signal was presented, and not to move when a NO-GO signal was presented.
https://doi.org/10.1007/s00406-004-0488-z	Asahi et al. (2004)	17	range 23-30 y.o.	10/7	325 ms	Nogo/Stop vs Go	11	The task consisted of eight alternating 36-s epochs of Go and No-Go conditions.Subjects viewed a series of letters once every 1500 ms and responded with a key press using the forefinger of the right hand to every letter except the letter 'X', to which they were instructed to withhold response. Stimulus duration was 500 ms and the interstimulus interval was 1000 ms.

https://doi.org/10.1016/j.neuropsychologia.2008.04.023	McNab et al. (2008)	14	range 22-34 y.o.	4/10	329 ms	Nogo/Stop vs Go	6	Go/No-Go task: 50% of trials involved the presentation of a yellow square for 1300 ms, followed by a blank screen for 400 ms, and a fixation cross for 300 ms (go trials). In response to presentation of the yellow square participants were required to press a button. For 25% of the trials the stimulus consisted of a yellow triangle which indicated that participants should not make a response (Stop/No/go trials). For the remaining 25% of trials the same presentation sequence was used, but the stimulus consisted of a blue square, which also required a button press. A stop-signal task: 50% of trials (control trials) involved the presentation of a yellow horizontal arrow, displayed for 1500 ms, followed by a blank screen for 400 ms and a fixation cross for 300 ms. The presentation of the yellow arrow required participants to press a button. In 25% of trials the yellow horizontal arrow was followed by a vertical yellow arrow, which signaled that the participant should inhibit their response (stop trials). In the remaining 25% of trials (oddball trials) the initial horizontal yellow arrow was followed by a second horizontal arrow, which was blue, and a button press was still required.
https://doi.org/10.1007/s00406-013-0483-3	Ko et al. (2014)	23	mean 24.35 y.o.	0/23	334 ms	Nogo/Stop vs Go	1	Three sections of the Go/No-go task with the same design, but a different sequence, were processed in this study. Each section consisted of 180 trials (150 go trials and 30 no-go trials) shown as numbers 1–9 with 200-ms durations and 1,425-ms inter-stimulus intervals. The participants were instructed to press the button as quickly as possible for all numbers (1, 3–9) except for the number 2. The task resulted in a total of 540 trials, lasting 932 s.
https://doi.org/10.1002/1097-0193(200102)12:2%3C100::aid-hbm1007%3E3.0.co;2-6	Liddle et al. (2001)	16	mean 30.2 y.o.	9/7	338 ms	Nogo/Stop vs Baseline	15	The presentation of each trial began with a descending series of asterisks. Participants were instructed to respond as quickly and accurately as possible with their right index finger every time ’X’ was presented and not to respond when ’A’ was presented. Each stimulus was presented for a period of 250 ms. A single run contain 24 Go and 24 No-go trials.
https://doi.org/10.1006/nimg.2002.1326	Garavan et al. (2002)	14	range 19-45 y.o.	10/4	338 ms	Nogo/Stop vs Baseline	16	Letters X and Y were presented serially in an alternating pattern at 1 Hz and subjects were required to make a button press response to each letter. Responses were to be withheld to lure stimuli: a lure occurred when the alternation was interrupted (e.g., the fifth stimulus in the train X-Y-X-Y-Y-X-Y). The event-related design of this experiment

								allowed the lures to be distributed unpredictably throughout the stimuli stream. Subjects completed four runs that contained 1180 targets (GO stimuli) and 80 lures (NOGO stimuli), resulting in an average interlure interval of 15.75 s.
https://doi.org/10.1016/s1053-8119(03)00334-3	Garavan et al. (2003)	16	range 18–46 y.o.	10/6	339 ms	Nogo/Stop vs Baseline	7	Letters X and Y were presented serially in an alternating pattern at 1 Hz and subjects were required to make a button press response to each letter. Responses were to be withheld to lure stimuli: a lure occurred when the alternation was interrupted (e.g., the fifth stimulus in the train X-Y-X-Y-Y-X-Y). The event-related design of this experiment allowed the lures to be distributed unpredictably throughout the stimuli stream. Subjects completed four runs that contained 1180 targets (GO stimuli) and 80 lures (NOGO stimuli), resulting in an average interlure interval of 15.75 s.
https://doi.org/10.1016/s0926-6410(03)00144-7	Mostofsky et al. (2003)	48	mean 27.45 y.o.	24/24	339 ms	Nogo/Stop	3	Cues consisted of drawings of green (Go-82%) and red (No-go) spaceships. Subjects were instructed to push the button as quickly as possible in response to green spaceships only and not to push during No-go signal. During each run the subject encountered 123 green cues, 27 red cues, and four long (10-s) rest phases (2 run).
https://doi.org/10.1162/jocn.2008.20100	Zheng et al. (2008)	18	range 22-40 y.o.	10/8	340 ms	Nogo/Stop vs Go	8	The number of trials in each run varied slightly across subjects to be around 104 (96–112). Both go trials and stop/no-go trials would finish at a button press or after 1000 msec had passed since they began. The task consisted of 75% go trials and 25% stop/no-go trials**.**
https://doi.org/10.1016/j.neures.2010.05.005	Yanaka et al. (2010)	27	mean 23.45 y.o.	13/14	342 ms	No go - Go	5	The subjects were initially presented with a central fixation cross. After a relatively long ITI of 12–14 s, the color of the fixation cross changed from white to yellow as a warning stimulus. Following a variable time period (2–6 s), a blue or red square was presented as the Go signal or NoGo signal, respectively. When the Go signal was presented, the subjects had to respond by pressing a button with their right thumb as quickly as possible. Duration was about 30 mins. Data were analyzed with GLM; . The VW task consisted of two successive experimental runs, which together comprised 10 Go trials and 10 NoGo trials.
https://doi.org/10.1016/j.schres.2007.07.033	Kaladjian et al. (2007)	21	mean 35.7 y.o.	2/19	343 ms	Nogo/Stop vs Go	11	A equiprobable Go/No-go task. After a series of cues which lasted 5 s, either the Go stimulus (letter ‘X’) or the NoGo stimulus (letter ‘A’) was presented for 250 ms. Participants were asked to respond to each presentation of the Go stimulus, by pressing a button, and to withhold response to each presentation of the NoGo stimulus. The task

								consisted of two runs lasting 11 min 08 s each. Each run contained 25 NoGo and 25 Go trials, presented in a pseudo-random order.
https://doi.org/10.1016/j.bbr.2017.06.030	Criaud et al. (2017)	20	range 20-42 y.o.	10/10	344 ms	Nogo vs Go	13	Participants were asked to react to visual go stimuli by pressing a button with the right thumb. At the beginning of a trial, participants saw a fixation point that could be red (main condition: indicated that either a go stimulus, a no-go stimulus or no stimulus at all could occur) or green (control condition). Each session consisted of 20 go trials, 20 no-go trials, 20 go_control trials and 20 catch trials (no stimulus), randomly presented, for a sum of 80 trials/condition of interest, giving a total of 320 trials for the experiment.
https://doi.org/10.1111/j.0953-816x.2004.03429.x	Kelly et al. (2004)	15	range 23 - 40 y.o.	10/5	346,9 ms	Nogo/Stop vs Go	23	The letters X and Y were presented serially in an alternating pattern and subjects were required to make a button press to each letter. Subjects were instructed to withhold a response to NOGO stimuli: an interruption to the alternating pattern whereby a letter was presented twice in a row. Stimulus durations were 700 ms and 1100 ms, followed by 100 ms ISI. The event-related design allowed the NOGO stimuli to be distributed unpredictably throughout the stimuli stream. Subjects completed four runs comprising 1152 GOs and 100 NOGOs.
https://doi.org/10.1162/08989290 4970726	Hester et al. (2004)	15	range 23–40 y.o.	10/5	355 ms	Nogo/Stop vs Go	21	Subjects were required to make a button press response to each letter (X or Y), which were presented at 1 Hz (900 msec on screen, 100-msec interstimulus interval). Responses were to be withheld to no-go events, which on 50% of occasions was preceded by a visual cue occurring two to seven trials in advance of the no-go event. Subjects completed 4 runs that contained 1176 targets (go stimuli) and 80 lures (no-go stimuli) that included an equal distribution of cued and uncued lures.
https://doi.org/10.1111/j.1399-5618.2009.00722.x	Kaladjian et al. (2009)	10	mean 41.5 y.o.	5/5	355 ms	Nogo/Stop vs Go	12	The presentation of each stimulus was preceded by an identical warning phase. Subjects had to respond pressing a button as quickly as possible to Go trials and to withhold response to NoGo trials. The Go and NoGo stimuli consisted of the letter 'X' and 'A', respectively. The task consisted of two runs lasting 11 min 08 s each. Each run contained 25 NoGo and 25 Go trials, presented in a pseudo-random order.
https://doi.org/10.1016/j.pscychr	Kaladjian et al. (2009)	20	mean 34.6 y.o.	10/10	356 ms	Nogo/Stopvs Go	16	The Go and NoGo stimuli consisted of the letter 'X' and 'A', respectively. Subjects were instructed to press a button as quickly as possible upon each presentation of the Go stimulus, and to withhold their response for

esns.2008.08.003								the NoGo stimulus. The task consisted of two runs lasting 11 min 08 s each. Each run contained 25 NoGo and 25 Go trials, presented in a pseudo-random order.
https://doi.org/10.1503/jpn.150335	Skunde et al. (2016)	29	mean 27.25 y.o.	not reported	357 ms	Nogo/Stop vs Go	25	The event-related design was presented in 2 runs, each containing 8 blocks. Participants were instructed to respond to the frequent target stimulus (square) but to inhibit any reaction to the rare nontarget stimulus (circle). In bothmodalities, each block consisted of 40 stimuli presented in a pseudorandom order with no-go stimuli occurring during 20% of the trials (40 x 8 blocchi x 2 sessioni = 640 stimoli totali)
https://doi.org/10.1111/pcn.12224	Chen et al. (2015)	15	mean 24.47 y.o.	0/15	360 ms	Nogo/Stop vs Go	7	In the Go condition, a numeral from 1 to 5 was shown 20 times in a white font on a black background. In the Nogo condition, a non- target (number 0) was shown 10 times, and a target (a number from 1 to 5) was shown 10 times in a pseudorandom sequence. The participants were told to press the button as quickly as possible for all numbers except for the number 0. The duration of the number presentation was 0.2 s and the inter-trial interval was 1.3 s.
https://doi.org/10.1016/j.jpsychires.2008.05.004	Mazzola-Pomietto et al. (2009)	16	mean 34.6 y.o.	10/6	360 ms	Nogo/Stop vs Go	7	A warned equiprobable Go/NoGo task. Two runs: each run contained 25 NoGo and 25 Go trials, presented in a pseudo-random order. Each trial began with a 5 s warning phase consisting of a countdown. Subjects were instructed to press a right-handed button box as quickly as possible upon the presentation of the letter 'X' and to withhold response on the appearance of the letter 'A'.
https://doi.org/10.1016/j.neuroimage.2016.05.061	Pornpattananangkul et al. (2016)	58	mean 23.25 y.o.	29/29	363 ms	Nogo/Stop vs Go	10	Participants were asked to respond with their right index finger to every letter except for the designated "nontarget" ("v" and "レ "). In the Go/No-Go condition were presented 10 targets and 10 nontargets. Each of 20 letters in each block was presented for 500 ms, followed by an inter-stimulus interval of 1 s, giving rise to a 30-s block. Blocks were separated by 20-s rest periods, where a fixation cross was shown; giving a total of 120 stimuli presentations during this 8-min task.
https://doi.org/10.1016/j.neulet.2017.08.031	Gavazzi et al. (2017)	26	mean 29.5 y.o.	15/11	364 ms	Nogo/Stop vs Baseline	11	Subjects were asked to press a button with their right index finger when a Go stimulus was presented. A descending series of asterisks was presented at the beginning of each trial for 250 ms, followed by a blank screen for 750 ms. The countdown continued until a single asterisk was displayed. Depending on the condition was displayed the Go ('X') or the Nogo ('A') stimulus for a temporal interval of 250 ms. The experimental session was composed by a total of 48 trials: 24 Go and 24 Nogo trials.

https://doi.org/10.1111/ejn.14301	Gavazzi et al. (2018)	36	mean 30.7 y.o.	21/15	365 ms	Nogo/Stopvs Baseline	20	Partecipants were asked to press a button as quickly as possible with their right index finger when a "Go" stimulus was presented and not to respond when "Nogo" stimulus was displayed. A descending series of points was presented at the beginning of each trial to prepare partecipants to the proper GNG stimulus ("readiness" period). Duration was about 12 mins.
https://doi.org/10.1162/jocn.2008.20100	Zheng et al. (2008)	18	range 22-40 y.o.	10/8	369 ms	Nogo/Stop vs Go	10	There were six 5-min runs in the Stop Signal Task scanning session. The number of trials in each run varied slightly across subjects to be around 104 (96–112). Both go trials and stop/no-go trials would finish at a button press or after 1000 msec had passed since they began. Both tasks consisted of 75% go trials and 25% stop/no-go trials.
https://doi.org/10.1016/j.kjms.2015.01.001	Chen et al. (2015)	25	mean 25.64 y.o.	0/25	370 ms	Nogo/Stop vs Go	7	Each section of the Go/No-go task consisted of 180 trials (150 Go trials and 30 No-go trials) shown as numbers 1-9 with 200 ms durations and 1425 ms interstimulus intervals. The participants were asked to press the button as quickly as possible for all numbers except for number 2. The task resulted in a total of 540 trials over 932 seconds.
https://doi.org/10.1016/j.jpsychires.2007.07.016	Karch et al. (2008)	16	mean 39.3 y.o.	0/16	370 ms	Nogo/Stop vs Baseline	13	The auditory stimuli consisted of sinus tonus (duration: 40 ms, pressure level: 100 dB) of three differential pitches. The tone with the middle frequency served as cue indicating that a button press was required when it was directly followed by the high frequency tone (go condition). The prepared behavioral response was to be inhibited if the cue was followed by the tone with a low frequency (nogo condition). One experimental run took about 25 min.
https://doi.org/10.1371/journal.pone.0020840	Cai & Leung (2011)	23	range 18-39 y.o.	11/12	374 ms	Nogo/Stop vs Go	21	After a 6.5-sec delay (black screen), a warning signal was presented for 1 sec followed by a response block of 9 trials. A go signal (500 ms) was presented on each trial. On some trials (about 30%), a stop signal (300 ms) was presented shortly after the go signal. The go signal was a black triangle and the stop signal was the same triangle encircled in a black circle. Trial durations varied between 1, 1.5 or 2 sec.
https://doi.org/10.1016/j.bbr.2013.06.001	Steele et al. (2013)	102	mean 33.92 y.o.	53/49	375 ms	Nogo/Stop vs Go	36	Two runs of 240 trials. Each stimulus appeared for 250 ms in white text. Participants were instructed to answer as fast as possible with the right index to the letter 'X' (Go signal) and to stop when the letter 'K' was presented (Nogo/Stop signal).
https://doi.org/10.1016/j.pscychresns.2015.09.017	van Eijk et al. (2015)	18	mean 25.28 y.o.	18/0	384 ms	Nogo/Stop vs Go	5	A stream of consonants was presented serially in the center of the screen. Every stimulus was displayed for 500 ms immediately followed by a blank screen for 500 ms. Subjects were instructed to make a right index finger button press for every

								letter except for the letter "X". Mean probability for nogo stimuli was 29%. Per run, 300 stimuli were presented.
https://doi.org/10.1523/jneurosci.4682-05.2006	Aron et al. (2006)	13	mean 29.2 y.o.	9/4	393 ms	Nogo/Stop vs Go	35	On each trial, a left- or right-pointing arrow stimulus was displayed on a computer screen. For the Go task, the subject responded as fast as possible with a left or right key press (using index and middle fingers of the right hand). For the Stop signal (25% of trials), the subject attempted to stop his/her response when a Stop signal was sounded at a particular SSD subsequent to the arrow stimulus. There were 32 Stop and 96 Go trials per scan (128 trials total).
https://doi.org/10.1161/strokeaha.118.022923	Gavazzi et al. (2018)	16	mean 38.3 y.o.	8/8	394 ms	Nogo/Stop vs Baseline	14	Participants were asked to press a button as quickly as possible with their right index finger when a "Go" stimulus was presented and not to respond when "Nogo" stimulus was displayed. A descending series of asterisks was presented at the beginning of each trial to prepare participants to the proper GNG stimulus ("readiness" period).Duration was about 12 mins.
https://doi.org/10.1002/1097-0193(200103)12:3%3C131::aid-hbm1010%3E3.0.co;2-c	Menon et al. (2001)	14	mean 23.6 y.o.	8/6	399 ms	Nogo/Stop vs Go	13	30-sec rest epoch, 12 alternating 26-sec epochs of Go and Go/NoGo conditions, followed by a 30-sec rest epoch. Subjects viewed a series of letters once every 2 sec and responded with a keypress to every letter (using the forefinger of the right hand) except the letter "X," to which they were instructed to withhold response.
https://doi.org/10.1016/j.pscychresns.2014.11.005	Penfold et al. (2014)	20	mean 35.67 y.o.	10/10	400 ms	Nogo/Stop vs Go	30	Stimuli consisted of a sequence of letters presented one at a time via in-scanner goggles. Subjects responded pressing a button whenever a letter other than "X" appeared on the screen. Following an initial 30-s rest block, there were eight alternating 30-s Go and NoGo blocks, with an additional 22.5-s rest at the end. Before each NoGo block a 2-s instruction screen ("Press for all letters except X")was presented. The letter "X" appeared randomly for 25% of trials. Stimulus presentation lasted 0.5 s with an inter-stimulus interval of 1.5 s.
https://doi.org/10.1111/ejn.12497	Hughes et al. (2014)	12	mean 27.25 y.o.	6/6	401 ms	Nogo/Stop vs Go	15	Each trial began with a visual stimulus (a single letter, O or X; 100 ms duration, 50% probability), separated by a stimulus onset asynchrony of 2000 ms. On 33% of trials, an auditory stimulus (1000 Hz, 100 dB square-wave tone of 50 ms duration) was presented after the onset of the visual stimulus. Go stimuli were presented within a small black square centred over a horizontal rectangle

								that differed in colour for each condition. Duration was about 8 min.
https://doi.org/10.1093/cercor/11.9.825	Braver et al. (2001)	14	range 18-27 y.o.	5/9	408 ms	Nogo/Stop	11	Subjects were instructed to withhold responding to infrequent No-go stimuli (the letter ‘X’ = 17% frequency) in the context of responding to frequent Go stimuli (the 25 ‘non-X’ letters = 83% frequency). Each stimulus appeared for 250 ms, followed by a 1000 ms inter-trial interval. Subjects responded by pushing a fiber optic light-sensitive key- press.
https://doi.org/10.1111/ejn.12425	Dambacher et al. (2013)	17	mean 28.4 y.o.	0/17	412 ms	Nogo/Stop vs Baseline	13	Participants were instructed to respond to a go stimulus via button press. Go as well as no-go stimuli were presented for 200 ms followed by a fixation cross for a randomized interval of 1300, 2800, or 4300 ms. No-go/stop events occurred in 25% of the 160 pseudo-randomized trials. The letters C and M were used as stimuli. Participants had to perform two consecutive runs of 160 trials in randomized order, giving a total of 320 trials (80 no-go/stop trials -approximately 10 min).
https://doi.org/10.1016/j.bbr.2013.08.026	Hughes et al. (2013)	15	mean 27.5 y.o.	8/7	413 ms	Nogo/Stop vs baseline	4	Stimulus sequences began with a 5 s countdown, followed by a block of 220 trials lasting 5 min and 30 s. Go task stimuli were the letters O and X, presented with equal probability. Stop-signals (1000 Hz, 50 ms, 85 dB) were presented during 30% of these trials (66 trials in each block) in a pseudo random fashion such that stop signal trials were always preceded by at least one go trial. Go stimulus onset asynchrony (including both go task and stop-signal task trials) was varied randomly (mean = 1.5 s), sampled from an exponential distribution.
https://doi.org/10.1162/jocn_a_01370	Yoon et al. (2019)	18	mean 30.7 y.o.	9/9	416 ms	Nogo/Stop vs Go	19	Trials begin with the appearance of a circular ring in the center of a black background screen. After 500 msec, an arrow pointing right or left, randomized with 50% probability, is shown within this ring. This indicates to the participant to respond with a right or left button press using the middle or index finger. The Stop trials are identical except for the presentation of an auditory stimulus (900 Hz, duration = 500 msec), which is presented after a short delay following the presentation of the arrow stimulus and signals the need to withhold the response. There were 64 Stop trials and 192 Go trials.

https://doi.org/10.1002/hbm.23230	Schmüser et al. (2016)	21	mean 35.00 y.o.	14/7	419 ms	Nogo/Stop vs Go	7	On the center of the screen a stream of in total 300 consonant letters was presented serially. Each consonant was shown for 500 ms followed by a black screen for the next 500 ms. The consonants were presented in a pseudo-randomized manner, with the restriction that the Nogo stimulus (X) is presented with a mean probability of 29% and each Nogo stimulus is followed by at least one go stimulus. During task performance, the subjects were instructed to respond by pressing a mouse button with the right index finger to every go stimulus and to withhold this response in case of the Nogo stimulus. In total, every subject completed two runs each consisting of 300 stimuli.
https://doi.org/10.1007/s11682-018-9868-2	Zhao et al. (2019)	20	mean 19.9 y.o.	9/11	433 ms	Nogo/Stop vs Go	48	Each run was composed by Go stimuli (75% of trials) and Stop stimuli (25% of trials). On each trial, a left- or right-pointing arrow was displayed on a computer screen. For the Go stimulus, subjects were instructed to press the left or right button as fast as possible. For the Stop stimulus, subjects attempted to stop pressing the button when the white arrow changed to blue. Each session consisted of two runs. Each run lasted for 4.5 min.
https://doi.org/10.1111/1469-8986.3720216	Khiel et al. (2000)	14	mean 28.4 y.o.	7/7	438 ms	Nogo/Stop vs Baseline	8	Participants were instructed to respond as quickly and accurately as possible with their right index finger every time the “X” appeared and not to respond to the “K”. Each stimulus run lasted for 423 s.
https://doi.org/10.1016/j.neuropsychologia.2014.07.033	Mulligan et al. (2014)	62	mean 18.01 y.o.	33/29	438 ms	Nogo/Stop vs Baseline	14	Participants saw a series of lower case letters presented on a computer screen replacing one another at a rate of 500 ms/letter. Participants were required to respond with a button press whenever specific target letters (“x” and “y”) were presented, but were required to withhold their response whenever nonalternating target letters were presented (“x” followed by “x” or “y followed by “y”). Six runs of this task (250 letters per run, 1500 total letters).
https://doi.org/10.1002/hbm.20564	Lawrence et al. (2008)	21	range 17.8-23.4 y.o.	9/12	440 ms	Nogo/Stop vs Go	2	Arrows of 500-ms duration appear on the middle of the screen and point to either the left or right side. After the 500 ms stimulus duration,there is a blank screen of 1.1–1.5 s, so that each inter-trial-interval amounts on average to 1.8 s. The participant is instructed to press the left or right response button as fast as possible,depending on the direction of the arrow. Infrequently (in 12% of trials), arrows pointing to the top (no-go signals) appear in the middle of the screen with 500 ms duration. Participants have to inhibit their response to these arrows. Task duration was 6.15 min.

https://doi.org/10.1093/cercor/bht304	Montojo et al. (2013)	30	mean 23.0 y.o.	18/12	440 ms	Nogo/Stop vs Go	5	Participants were instructed to respond quickly when a “go” stimulus was presented on the computer screen (which consisted of leftward or rightward pointing arrows), except on the subset of trials where the “go” stimulus was followed by a “stop” signal (a 500-Hz tone presented through headphones).
https://doi.org/10.1016/j.pscychresns.2009.05.002	Page et al. (2009)	11	mean 34.1 y.o.	0/11	443 ms	Nogo/Stop vs Baseline	11	Arrows in the centre of the screen pointed either left or right for 300 ms and the subject had to press the left or right button, respectively, during the remaining 1.5 s of blank screen. Pseudo- randomly, No-go arrows pointing upwards appeared on the screen (not at the same time as the Go arrows) and the subject was instructed not to respond to these. Each task lasted about 6 min.
https://doi.org/10.1016/j.neures.2011.03.007	Tabu et al. (2011)	13	mean 27.5 y.o.	5/8	445 ms	Nogo/Stop vs Go	6	On each trial, after a fixation period the central fixation point was replaced by a left- or right- pointing arrow in green color (Go cue) on a computer screen. Subjects were instructed to press a button using the right or left thumb, in response to the Go cue. In 25% of trials (Stop trials), a red rectangle was presented shortly after the presentation of a Go cue. There were 30 Stop and 90 Go trials per session. The number of leftward and rightward pointing arrows was equal.
https://doi.org/10.1016/j.neuroimage.2011.04.023	Schulz et al. (2011)	16	mean 23.6 y.o.	8/8	449 ms	Nogo/Stop vs Baseline	8	Each trial started with a cue presented for 250 ms, followed by a 2250 ms interval, after which the target was presented for 250 ms. There were two cue conditions: relax cue and ready cue, including right and left cues. There were also two target conditions: no-target and target, including go and no-go targets. Participants had to prepare to respond in the direction of the arrow cue and press the appropriate button for the green circles and withhold responses for the red circles. The average trial duration was 6 s.
https://doi.org/10.1007/s00213-013-3038-4	Kareken et al. (2013)	13	mean 23.7 y.o.	7/6	451 ms	Nogo/Stop vs Go	5	Each of 3 individual task runs consisted of 80 ‘Go’ trials, each requiring a left or right button press on a response box to horizontal green arrows pointing either left or right. 40 ‘Stop’ trials were marked by a red up-pointing arrow immediately after a Go stimulus (total of 240 Go trials and 120 Stop trials).
https://doi.org/10.1073/pnas.1000175107	Sharp et al. (2010)	26	range 23-59 y.o.	9/17	452 ms	Stop vs Go	10	A fixation cross was presented initially for 350 ms followed by the go stimulus for 1,400 ms (a right- or left-pointing arrow). Finger presses were made with the index finger of each hand. 20% of the trials involve an unpredictable stop signal (red dot) presented at a variable delay following the go signal.

https://doi.org/10.1002/hbm.20237	Rubia et al. (2006)	23	range 20-43 y.o.	Not reported	457 ms	Nogo/Stop vs Go	11	Arrows (500 ms) pointing either to the left or right side appear on the middle of the screen. Then, there is a blank screen of 1300 ms, so that each intertrial interval amounts to 1.8 s. The subject is instructed to press the left or right response button as fast as possible,depending on whether the arrow points left or right. Infrequently (in 12% of trials), arrows pointing to the top (no-go signals) appear in the middle of the screen with a 500-ms duration. Subjects have to inhibit their motor response to these arrows. Task duration was just over 6 mins.
https://doi.org/10.1016/j.biopsycho.2011.10.013	Hughes et al. (2012)	10	mean 35.1 y.o.	3/7	457 ms	Stop>Baseline	5	Go stimuli were the letters 'O' and 'X' presented equiprobably and mapped to left and right thumb-press responses, respectively. On go trials (75%), a single go stimulus was presented. On stop-signal trials (25%), the go stimulus was followed by an auditory stop-signal (1000 Hz, 50 ms, and 85 dB). Trials were presented at a constant 2000 ms inter-trial interval in blocks of 224 trials lasting 7 min 28 s.
https://doi.org/10.1073/pnas.96.14.8301	Garavan et al. (1999)	14	mean 31 y.o.	6/8	460 ms	Nogo/Stop	14	Subjects made a button response, with alternation, to the target letters X and Y, and withheld response on nonalternating presentations of these target letters. Letters were presented for 500 msec in black against a white background with 0-msec interstimulus interval. In total, there were 150 targets and 25 lures.
https://doi.org/10.1016/j.neuroimage.2019.04.021	Weafer et al. (2019)	44	mean 24.7 y.o.	22/22	462 ms	Nogo/Stop vs Go	15	Participants were instructed to respond as quickly as possible to go signals (left- or right-pointing blue arrows), and to inhibit responses on trials in which a stop signal (up-pointing red arrow superimposed on the go arrow) occurred. Participants completed three task runs (80 go and 40 stop trials each), and each run required approximately 6 minutes to complete.
https://doi.org/10.1162/jocn_a_00567	White et al. (2014)	123	mean 31.14 y.o.	57/66	488 ms	Nogo/Stop vs Go	6	Participants were shown a series of Go stimuli (left- and rightwards pointing arrows) in the center of the screen and were told to respond with left and right button presses (Go trials). On a subset of trials (25%), a stop-signal (a 500 Hz tone) was presented a short delay after the Go stimulus appeared and lasted for 250 ms (Stop trials). Each experiment block consisted of 128 trials, 96 of which were Go trials and 32 of which were Stop trials. Participants completed a total of two blocks for a total of 256 trials.
https://doi.org/10.1016/j.bbr.2020.112586	Gaillard et al. (2020)	38	mean 26.63 y.o.	23/15	489 ms	Nogo/Stop vs Go	5	Participants completed two stimulus blocks including 140 trials (100 go trials, 40 stop trials) that were presented pseudorandomly. Go trial stimuli were left-/right-pointing arrows, presented centrally on a grey background, that each appeared on screen

								for 1 s. Participants were instructed to make speeded responses with either the right index finger (left pointing arrow) or right middle finger (right pointing arrow) using a button box and to stop their response when a stop signal sounded.
https://doi.org/10.1016/j.neuroimage.2011.03.053	van der Meer et al. (2011)	19	mean 21.6 y.o.	9/10	490 ms	Nogo/Stop vs Go	13	A stop-signal task. Go conditions: subjects simply pressed the button to the left or right upon presentation of the stimulus (arrows pointing left or right). Stop-condition: an arrow pointing upward was presented beside the arrow pointing left or right and appeared either just after or simultaneously with the first arrow. In this case, subjects should hold back their response. A total of 160 go trials and 40 stop trials.
https://doi.org/10.1016/j.pscychresns.2015.09.017	van Eijk et al. (2015)	18	mean 25.28 y.o.	18/0	493 ms	Nogo/Stop vs Go	7	A white arrow appeared within the fixation ring and the subjects were instructed to respond corresponding to the pointing direction of the arrow (go condition). In 25% of the trials the fixation ring changed color after a variable delay time and subjects were instructed to attempt to cancel the reaction in such a case (stop condition). Participants performed two runs (128 trials each one) of the task.
https://doi.org/10.1016/j.cortex.2016.12.012	Kolodny et al. (2017)	20	mean 27.4 y.o.	13/7	516 ms	Nogo/Stop vs Go	5	Participants were instructed to respond when a Go stimulus (a red square) was presented and to withhold response to all other stimuli (squares in other colors,red shapes other than squares, or other shapes in other colors). In the rare-No-go condition, 75% of trials were Go trials and 25% were No-go trials. Each stimulus was presented centrally on its own for 100 msec, and the inter-stimulus- interval (ISI) varied with a mean of 2.75 sec. Each block consisted of 164 trials, and lasted a total of 8 min.
https://doi.org/10.3389/fnhum.2014.00027	Schel et al. (2014)	24	mean 21.49 y.o.	13/11	519 ms	Nogo/Stop vs Go	13	Each trial started with the presentation of a green left- or rightwards pointing arrow: participants were instructed to respond to the direction of the arrow pressing a button with their left or right index finger. On a limited number of stop-trials (25%) a stop-signal was presented (the arrow suddenly changed color to red). The experiment consisted of two blocks of 128 trials, each block consisting of 96 go-trials and 32 stop-trials.
https://doi.org/10.1016/j.neuroimage.2014.01.023	Baumeister et al. (2014)	23	mean 24.70 y.o.	11/12	544 ms	Nogo/Stop vs Go	16	Stimuli consisted of an array of five shapes including a central target arrow pointing either left or right, flanked by two shapes (arrows, squares or Xs) on each side. Subjects were instructed to press a button corresponding to the central arrow when the flankers were also arrows or boxes, but not when they were Xs. Duration was about 10 mins.

https://doi.org/10.1016/j.biopsych.2006.12.007	Roth et al. (2007)	14	mean 34.9 y.o.	8/6	547 ms	Nogo/Stop vs Go	13	Participants were required to press a button with the index finger of their right hand when they saw a circle with a vertical cross inside and withhold responding when they saw a circle with an X inside. Stimuli were presented in a single run of approximately 5-min duration. The run consisted of three task epochs of 24 stimuli each (for a total of 36 go and 36 no-go stimuli).
https://doi.org/10.3389/fnhum.2015.00034	Xu et al. (2015)	18	mean 26.4 y.o.	9/9	572 ms	Nogo/Stop vs Go	14	The stimuli consisted of four orientations of an arrow with a fixation point "+" in the middle. The arrow stimuli were presented one at a time for a duration of 1500 ms or until a response was made. All responses were made with the right index finger. The primary response required pressing the button consistent with the arrow orientation. The Stop condition included a delayed visual-cue.
https://doi.org/10.1016/j.neuropsychologia.2019.107220	Messel et al. (2019)	28	mean 24.68 y.o.	20/8	572 ms	Nogo/Stop vs Go	8	The task consisted of 600 task-trials in total: these were divided over three different cue conditions: 0%, 25% and 66%. Go-signals consisted of either a left- or right-ward pointing green arrow, indicating the response-hand to be used. In stop-trials, a blue arrow pointing in the same direction as the preceding go-signal was presented. 750 trials (task and null-event trials) were divided into 10 experimental blocks, the overall duration of the task was approximately 1 h.
https://doi.org/10.1038/oby.2011.180	Hendrick et al. (2012)	43	mean 31.4 y.o.	43/0	600 ms	Nogo/Stop vs Go	43	A dot appeared on the screen at the beginning of a go trial, after a randomized time interval the dot turned into a circle (the "go" signal), prompting the subjects to quickly press a button. One quarter of all trials were stop trials. In a stop trial, an additional "X" appeared after and replaced the go signal. The subjects were told to withhold button press upon seeing the stop signal. Each subject completed four 10-min runs of the task.
https://doi.org/10.1016/j.bandl.2014.03.003	Rodríguez-Pujadas et al. (2014)	33	mean 21.205 y.o.	15/18	654 ms	Nogo/Stop vs Go	7	Two types of trials were employed in the task: Go trials (p = .75) and Stop trials (p = .25). In Go trials, participants responded manually (with the index finder or thumb)to the visual stimulus presented on the screen. In Stop trials, they were instructed to stop their manual response when a stop signal (beep) was sounded after the presentation of the visual stimulus. There were three runs in total with 96 Go trials and 32 Stop trials in each run.
https://doi.org/10.1002/hbm.23338	Leunissen et al. (2016)	22	mean 23.5 y.o.	11/11	816 ms	Nogo/Stop vs Go	22	The visual display consisted of a vertical indicator that moved from the bottom upwards on each trial. A target line was situated 800 ms from onset. Go trials were to stop the indicator at the target by releasing the switch. Sometimes the indicator stopped

								automatically prior to the target. When this happened, participants tried to prevent releasing the switch (Stop trials). The bar stopped automatically on 20% or 40% of the trials. Three scanner runs were completed (506 trials in total).
https://doi.org/10.1002/hbm.22047	Zandbelt et al. (2013)	22	range 20-28 y.o.	13/9	819 ms	Nogo/Stop vs Baseline	7	Participants are instructed to respond when a moving indicator reaches a target, but to suppress a response when this moving indicator stops automatically before reaching this target. Stop-signal probability was 0%, 24%, or 35%. Duration was about 42 mins.
https://doi.org/10.1016/j.neurobiolaging.2016.06.007	Bloemendaal et al. (2016)	48	mean 45.15 y.o.	20/28	847 ms	Nogo/Stop vs Go	13	Information load increased with level. Stop-signal probability increased as a function of cue color. Every level contained 70 trials with 0% (green) and 270 trials with >0% (white) stopsignal probability. Duration was about 38 mins.

Sensitivity Analysis Results

Table S3. Sensitivity analysis.

Results from ALE meta-analysis. MNI coordinates. BA = Brodmann's area.

CG-Go/NoGo subsample: ALE metanalysis computed from our study selection

Cluster	x	y	z	ALE	P	Label (Nearest Gray Matter within 5mm)
1	32	24	-6	0.024634913	2.81E-08	Right Claustrum
	44	30	-10	0.016408807	1.68E-05	Right Inferior Frontal Gyrus.BA 47
	32	14	-10	0.008899848	0.002954644	Right Claustrum
2	36	40	26	0.015126572	4.42E-05	Right Superior Frontal Gyrus. BA 9
	36	50	26	0.014915977	5.20E-05	Right Superior Frontal Gyrus. BA 9
	42	34	26	0.014061867	1.00E-04	Right Middle Frontal Gyrus.BA 9
	28	52	42	0.009665802	0.001798898	Right Superior Frontal Gyrus.BA 8
	20	52	32	0.008833956	0.003086074	Right Superior Frontal Gyrus.BA 8
	26	50	32	0.008613995	0.003581225	Right Superior Frontal Gyrus. BA 9

IG-Go/NoGo subsample: ALE metanalysis computed from our study selection

Cluster	x	y	z	ALE	P	Label (Nearest Gray Matter within 5mm)
1	38	18	-8	0.021293892	2.93E-07	Right Insula.BA 13
	30	22	4	0.01169453	3.16E-04	Right Claustrum
	52	16	-4	0.010143784	7.73E-04	Right Insula.BA 13
	18	16	-8	0.009831957	9.42E-04	Right Putamen
2	-4	30	20	0.016219022	1.33E-05	Left Anterior Cingulate.BA 24
	6	10	50	0.015761267	1.92E-05	Right Medial Frontal Gyrus.BA 6
	0	20	40	0.013712626	8.90E-05	Left Cingulate Gyrus.BA 32
	-4	26	30	0.010149372	7.68E-04	Left Cingulate Gyrus.BA 32
	-6	12	42	0.008577168	0.002432727	Left Cingulate Gyrus.BA 32
3	-12	-8	18	0.019176658	1.36E-06	Left Caudate
	-12	6	12	0.017733935	3.92E-06	Left Caudate
4	-48	-2	46	0.01901833	1.53E-06	Left Precentral Gyrus.BA 4

Table S3. Results from ALE meta-analysis of both CG-Go/NoGo and IG-Go/NoGo subsamples.

From left to right, the table reports the number of clusters, stereotaxic MNI coordinates of local maxima, ALE scores, P scores and anatomical labelling (with corresponding Brodmann area) of the clusters that were consistently associated with successful inhibition of both CG-Go/NoGo and IG-Go/NoGo subgroups. - P < .01 cluster-level corrected inference using P < .005 uncorrected at voxel-level as the cluster-forming threshold generated by 2000 random permutation tests.

Table S4. Sensitivity analysis.

Results from ALE meta-analysis. MNI coordinates. BA = Brodmann's area.

Contrast CG-Go/NoGo – IG-Go/NoGo: ALE metanalysis computed from our study selection

Cluster	x	y	z	P	Label (Nearest Gray Matter within 5mm)
1	25	50	36	6.00E-04	Right Superior Frontal Gyrus.BA 8
	24	52	37	1	Right Superior Frontal Gyrus.BA 8
	27	52	30	0.0023	Right Superior Frontal Gyrus.BA 9

Conjunction IG-Go/NoGo & CG-Go/NoGo samples: ALE metanalysis computed from our study selection

Cluster	x	y	z	ALE	Label (Nearest Gray Matter within 5mm)
1	38	20	-10	0.014739954	Right Inferior Frontal Gyrus.BA 47
1	32	24	2	0.010000105	Right Claustrum
1	32	14	-10	0.008899848	Right Claustrum

Table S4. Results from ALE meta-analysis of both CG-Go/NoGo - IG-Go/NoGo and IG-Go/NoGo - CG-Go/NoGo samples contrast, and CG-Go/NoGo & IG-Go/NoGo samples Conjunction.

From left to right, the table reports the number of clusters, stereotaxic MNI coordinates of local maxima, ALE scores, P scores and anatomical labelling (with corresponding Brodmann area) of the clusters that were consistently associated with successful inhibition of both conjunction and contrasts analyses. The parameters of contrast analyses were set as an uncorrected p < .01 with 10,000 permutations.

Publication bias assessment

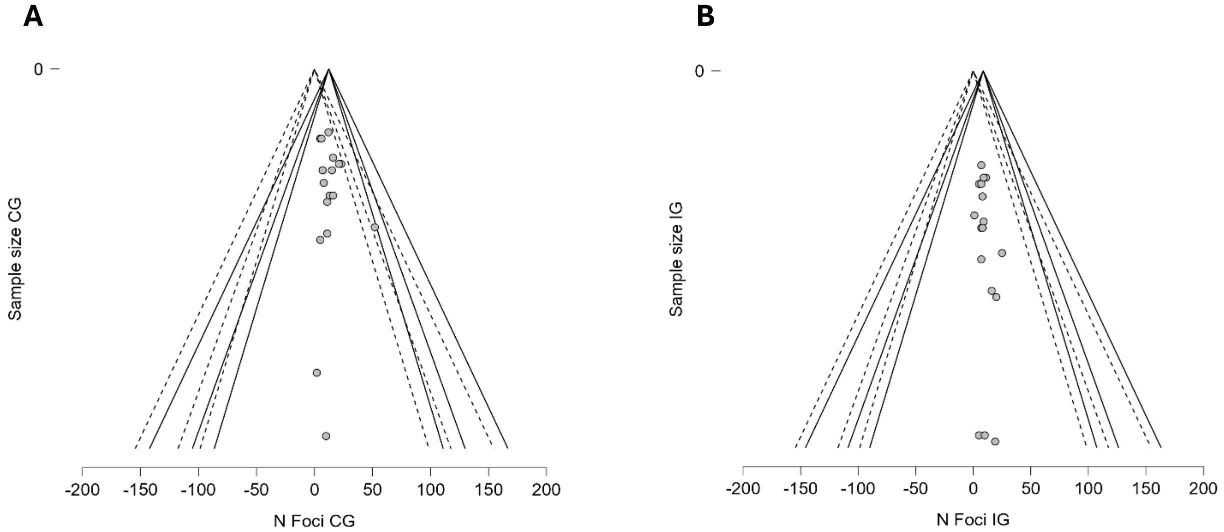

Figure S1. Funnel plots for publication bias assessment.

Funnel plots for the two groups of studies CG (Panel A) and IG (Panel B) included in the meta-analysis. The number of foci reported in each study is plotted on the x-axis, while the sample size is plotted on the y-axis. Visual inspection did not reveal any marked asymmetry, suggesting no evidence of substantial publication bias.
